# Supplementary material for: Mining GWAS and eQTL data for CF lung disease modifiers by gene expression imputation
Source: PLoS One. 2020 Nov 30;15(11):e0239189. doi: 10.1371/journal.pone.0239189 (PMC7703903; doi:10.1371/journal.pone.0239189)
Supplement: S4 File — (DOCX) [file pone.0239189.s004.docx]

**S1 Methods**

**GWAS imputation data set**

Newly imputed GWAS data set in VCF format [1] from the International Cystic Fibrosis Gene Modifier Consortium [2], were updated to current dbSNP v150 common variance annotations based on hg19 reference genome coordinates and REF/ALT alleles. For predictive model training with our own expression data, the imputed GWAS coordinates were also updated to reference genome hg38 (GRCh38p7) by cross-referencing dbSNP v150 downloads (<ftp://ftp.ncbi.nih.gov/snp/>), common_all_20170710.vcf.gz mapped to hg19 (GRCh37p13) and hg38 reference genomes, as of 07/2017. Imputed dosage of the ALT alleles of bi-allelic SNPs were parsed out from the GWAS VCF files and used to calculate minor allele frequencies and saved as genotype input for gene expression imputation.

**Gene expression from CF nasal scrape epithelial biopsies by RNA-seq**

The paired-end RNA sequencing reads of 49 bp from CF nasal mucosal epithelial biopsies [3], were re-mapped to hg38 reference genome, and ENSEMBL v90 gene and transcript annotation, downloaded from <http://aug2017.archive.ensembl.org/info/data/ftp/>, using HISAT2 v2.1.0 alignment program [4]. Quantification of gene expression were obtained using the assembler software, StringTie v1.3.3 [5]. The resultant fragments per kilobase per million (FPKM) mapped reads at gene level, were quantile normalized and log2 transformed after adding an offset value of 0.005 to generate normalized expression values. The normalized expression was regressed against selected clinical and technical variables using linear models as reported before [3], and the residuals were used as expression input for predictive model training. To reduce reference allele bias during short sequence read mapping [6], an alternative mapping by HISAT2 against both hg38 primary and alternative assemblies, incorporating common variance information from dbSNP v150, and Gencode v27 (equivalent of ENSEMBL v90) gene and transcript annotation, were performed. Gene expression quantification by StringTie and normalization were performed similarly as above. Gene expression values of comparable genes between primary and alternative assemblies were combined to represent the final gene expression values with ENSEMBL gene_id from the primary assembly as the unique identifiers for genes.

**Gene expression from CF lymphoblastoid cell lines (LCLs) by Affymetrix HuEx_1.0 exon microarrays**

Probe signals from CEL files corresponding to GEO data set, GSE60690 [7], were normalized to ENSEMBL v90 gene level expression values using the command line Affymetrix Power Tools (APT) v1.20.6 (<https://www.thermofisher.com/us/en/home/life-science/microarray-analysis/microarray-analysis-partners-programs/affymetrix-developers-network/affymetrix-power-tools.html>), and a custom meta-probeset mapping based on ENSEMBL expression microarray annotation pipeline (<http://aug2017.archive.ensembl.org/info/genome/microarray_probe_set_mapping.html>). Briefly, probesets mapping to ENSEMBL v90 were downloaded through Biomart (<http://aug2017.archive.ensembl.org/biomart/>), and a custom meta-probeset file was generated by consolidating Affymetrix HuEx-1.0st_v2.0 array transcript probesets mapped to target ENSEMBL Gene IDs. The custom meta-probeset file and the standard library files for the specific microarray platform were then used to derive log2 normalized expression values by the *apt-probeset-summarize* program in APT, with the “rma-sketch” workflow, which performs rma background correction, quantile normalization, and median polish summarization of probe intensities. The log2 rma expression values were then quantile normalized among the samples, regressed against selected clinical and technical variables as described before [7], and the residuals were used as gene expression input for predictive model training.

**HLA gene expression counts from AltHapAlignR**

To assess allele bias of HLA genes, gene expression counts from uniquely mapped sequence reads of HLA genes as defined by the international ImMunoGeneTics project (IMGT: <http://hla.alleles.org/genes/index.html>), were obtained from the alternative mapping alignment files above as outlined by AltHapAlignR [6]. Briefly, sequence reads mapped to chr6 HLA region, and chr6 HLA alternative assemblies (GL000250.2 to GL000256.2), as well as unmapped were extracted from the alignment BAM files, and re-mapped to the 7 alternative chr6 HLA assemblies separately by HISAT2. Uniquely mapped reads were compiled using *make_a_table.py* script, and counted by the *getMappingRatesFromPairs* R function from AltHapAlignR (<https://github.com/jknightlab/AltHapAlignR>). To obtain gene expression counts from StringTie quantification for comparison to AltHapAlignR, the *prepDE.py* script was used, which extracts read count based on StringTie coverage values. Since StringTie uses a network flow algorithm and de novo assembly to construct transcripts [5], the gene count data is not directly comparable to AltHapAlignR, therefore all comparisons are based on correlation among the same samples.

**Predictive model building using PredictDB_Pipeline**

Predictive models of genetic regulation of gene expression by genotype data, were generated using the training data sets of CF nasal epithelium and CF LCLs where tissue specific gene expression, and genotype data were available from the same patients, through modifications of the PredictDB_Pipeline_GTEx_v7 (<https://github.com/hakyimlab/PredictDB_Pipeline_GTEx_v7>) made available from the Im lab at the University of Chicago (<http://hakyimlab.org/>). The modifications involved updating directory and file names to point to the correct files locally. Specifically, the data sets were first updated to current reference genome GRCh38/hg38, dbSNP v150, and ENSEMBL v90 gene annotations, before used as input training data to the PredictDB pipeline. The core of the model building with the training data, is that genotypes of *cis*-SNPs within 1 megabase (Mb) flanking a gene were used as independent variables in an additive linear model for the normalized residuals of expression values of the gene, using the cross-validation procedure of penalized linear model fitting at elastic-net penalty of alpha = 0.5 with the R package, *glmnet* [8, 9]. The resultant models saved in SQLite3 database files, were filtered at prediction R^2^ > 0.01 and p-value < 0.05 for performance quality measures, as outlined by the Im lab (<http://hakyimlab.org/post/2017/v7-v6p-analysis/>) as of 01/2018. The results are predictive models where the genetically regulated gene expression is a linear function of selected *cis*-SNPs with optimized weights by elastic net regression from *glmnet* [8], that can be used to impute expression from the larger CF cohorts given genotype data. The numbers of genes that passed the predictive model performance filters, referred to as imputable genes, vary from tissue to tissue largely dependent on the sample size and gene expression quality [10].

**Pre-built predictive models from GTEx Consortium data release v7, and the Depression Genes and Networks (DGN; whole blood)**

Predictive models were downloaded from Im lab PredictDB data repository (<http://predictdb.hakyimlab.org/>), which were trained using RNA-seq expression and genotype data release v7 from the GTEx consortium [11], and DGN [12] as of 01/2018. The genotype data was updated to dbSNP v150 and filtered by HapMap CEU population (<https://s3.amazonaws.com/predictdb2/gtex_v7_hapmapceu_dbsnp150_snp_annot.txt>), and the gene expression data were based on Gencode v19 gene and transcript annotations against hg19 reference genome, available for download from GTEx portal (<https://www.gtexportal.org/>). All downloaded data were current as of 01/2018.

**Gene expression imputation from CF GWAS data**

Update GWAS imputation [1], in the form of SNP alt allele dosages, from CF patients who participated in the International CF Gene Modifier Consortium lung disease severity studies [13, 14], were formatted as described above and used as input for gene expression imputation for the entire CF GWAS cohorts, using the script, *PrediXcan.py* from the Im lab (<https://github.com/hakyimlab/PrediXcan>), as originally reported [8]. GWAS analysis at single SNP level from the updated GWAS imputation [1], were reproduced as meta-analysis of cohort/platform combinations according to published protocol [13]. These CF lung disease association p-values at single SNP level for SNPs used in gene expression predictive models were used to illustrate relationships between GWAS signals and imputed gene expression associations. Regional association p-values and linkage disequilibrium (LD) of SNPs from GWAS analysis were plotted using the LocusZoom plot [15]. GWAS p-values of relevant SNPs were formatted as bedGraph files, and visualized on the UCSC genome browser (<http://genome.ucsc.edu/>), as custom annotation tracks against appropriate reference genome versions. All downloaded software tools and data were current as of 12/2018.

**CF disease phenotype association testing for imputed gene expression**

The genetically regulated gene expression data imputed from CF GWAS cohorts using predictive models as described above were filtered to form a set of unrelated patient cohort, whose CF disease phenotypes were well characterized. Association of the imputed gene expression to disease phenotype were tested using linear models with the Bioconductor R package, *limma* [16], and the robust regression method instead of ordinary least squares regression. The robust regression is done by iterated re-weighted least squares with the *rlm* function from the R package, *MASS*, which is less influenced by extreme values [17, 18]. For disease phenotype association testing using predictive models trained on CF nasal epithelial biopsy, and LCL data sets, the samples used in predictive model training were excluded in the association testing.

For hierarchical clustering analysis, signed -log10p-value with direction of association beta coefficient as indicator of expression change direction were compiled for genes significantly associated to disease phenotype from multiple tissue data sets. Due to the uneven nature of number of imputable genes from different tissues, the compiled matrix contains >50% missing values. Distances between genes (rows) and tissues (columns) were calculated using the *dist()* function in R with Canberra distance metric, and the missing values in the resultant distance matrix were replaced with the largest distance value between any pairs (since the next step requires all numeric matrix). The final distance matrix were used to cluster genes and tissues using the *hclust()* function in R with “Ward.D2” method. Clustering heatmaps were generated using the Bioconductor R package, *ComplexHeatmap* [19]. Manhattan plots of GWAS data and imputed gene expression phenotype associations were generated using the R package, *qqman* [20], or with the *ggplot2* [21] and *ggrepel* packages.

**FUSION/TWAS imputation and functional analysis from CF GWAS summary data**

FUSION is an alternative method to gene expression imputation, that uses only GWAS summary statistics to perform transcriptome-wide association testing, or TWAS [10]. The software is available from the Gusev lab (<http://gusevlab.org/projects/fusion/>). Reference LD data based on 1000 genomes project EUR population was downloaded from <https://data.broadinstitute.org/alkesgroup/FUSION/LDREF.tar.bz2>, while predictive models from 48 human tissues based on GTEx v7 were downloaded from <http://gusevlab.org/projects/fusion/weights/GTEX7.txt>. All software and reference data were current as of 12/2018. GWAS signed z-scores from meta-analysis were compiled using the *munge_sumstats.py* script [22], and tested for association to disease phenotype according to the tutorial by the Gusev lab (<http://gusevlab.org/projects/fusion/>).

**Meta-analyses combining dependent multiple tissue p-values**

To combine phenotype association results from gene expression imputation of multiple tissues, meta-analyses combining p-values from correlated data sets were performed. The harmonic mean p-value (HMP) [23] method, which uses just the p-values from individual tests, was used to combine multiple p-values for both the PrediXcan and TWAS data results separately. In addition, an empirical adaptation of Brown’s method (EBM) [24], informed by imputed gene expression correlation from >500 randomly selected patients, was used as an alternative method for PrediXcan tests; while similarly, the omnibus test from the *FUSION.post_process.R* script of TWAS, which uses SNP LD information, was used to combine p-values informed by correlation from TWAS results. Unless otherwise indicated, the p-value < 0.01 was used as a threshold for significant association.

**Comparison between phenotype association testing results**

To assess correlation between different test results among multiple genes, simple linear regression was performed between 2 sets of test statistics, such as mean effect-sizes, or -log10 p-values from PrediXcan and TWAS meta-analyses, or GWAS.

**Genome-wide heritability estimation from CF GWAS imputation data**

Narrow-sense heritability (*h^2^*), or additive effects of SNPs, of CF lung disease phenotype (KNoRMA) from the imputed GWAS data was estimated using the Genome-wide Complex Trait Analysis (GCTA) following the GREML-LDMS tutorial from GCTA documents (<https://cnsgenomics.com/software/gcta/#GREMLinWGSorimputeddata>). Briefly, imputed GWAS genotype data [1] in PLINK format were used to derive segment-based LD scores using GCTA, and ~8.3 million SNPs were stratified by LD scores into 4 sets by quantiles of their LD scores. Each set of SNPs was used to derive genetic relationship matrix (GRM) among all the patients and combined into one GRM. Related patients were filtered at cryptic relatedness score of 0.025, and the final GRM of unrelated patients was used to calculate heritability of CF lung disease phenotype with sex and 4 genotype PCs as covariates.

**Gene set and pathway enrichment analysis**

Gene set enrichment analysis with pre-ranked protein coding genes were performed against selected collection of gene sets and pathways, using the Bioconductor R package, *fgsea* [25]. Specifically, protein coding genes from combined CF lung disease association testing from multiple tissues were ranked by the -log10 of the maximal p-value between the 2 meta-analyses for each platform. Collection of gene sets or pathways were tested for enrichment from the ranked genes using *fgsea* with 1 million permutations. The gene set collections used are Gene Ontology (GO) [26] with UniProt annotation (<https://www.uniprot.org/>), current as of 02/2019; pathways collections at the NCBI biosystems database [27], which contains information from source databases, such as KEGG, Reactome, Pathway Interaction Database, and WikiPathways, current as of 04/2017. These collection of gene sets and pathways were formatted as .gmt files [28] and imported into *fgsea*. Only gene sets containing minimum of 5 protein-coding genes, and maximum of 500 genes in each collection were tested for enrichment.

**Supporting Figures**

**
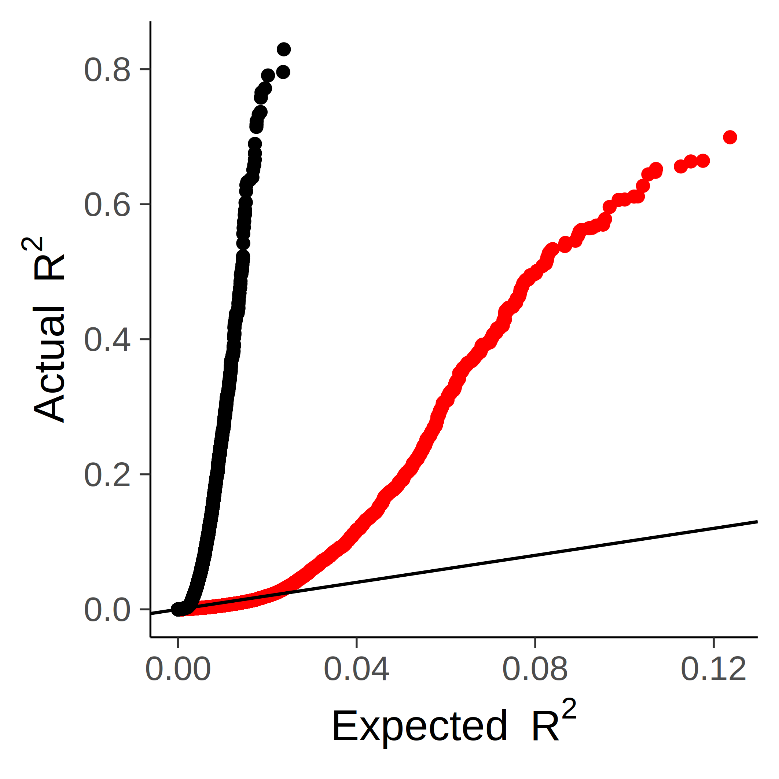
**

**S1 Fig**. **Correlation between predictive modeling and observed gene expression compared to null distribution, reflecting performance of the predictive models.** The expected R^2^ from null distribution on x-axis were plotted against the actual R^2^ from individual genes of predictive models on y-axis. The solid line represents null distribution of R^2^ derived from Fisher’s approximation, while significant deviation of data points above represents predictability of gene expression from genetic variants. Red dots denote R^2^ values from 132 CF nasal epithelial biopsy data set, while black dots represent similar values from 753 CF LCL data set.


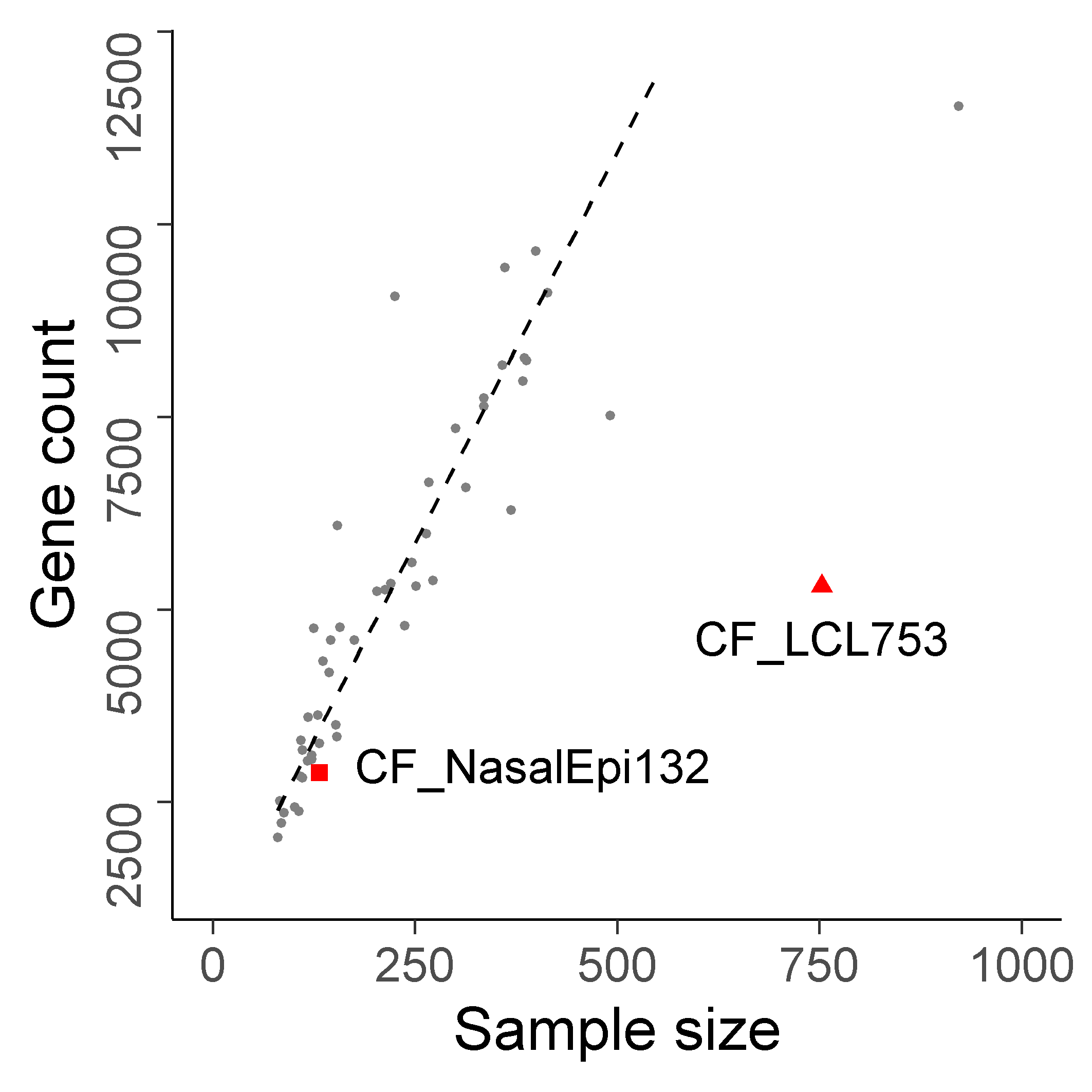


**S2 Fig**. **Correlation between training sample size and number of predictable genes.** The sample sizes retrieved from GTEx RNA-seq gene expression training data, DGN whole-blood, and CF gene expression data sets were plotted against the number of genes that passed predictive model thresholds (i.e. correlation between predicted and observed gene expression) of R^2^ > 0.01 and p-value < 0.05. The dashed line denotes linear robust regression (R package, *robust*) which is less influenced by extreme outlier data points. The CF LCL data set (red triangle, n=753) is based on microarray gene expression assay, while the CF nasal epithelial biopsy data set (n=132) is shown as red square.


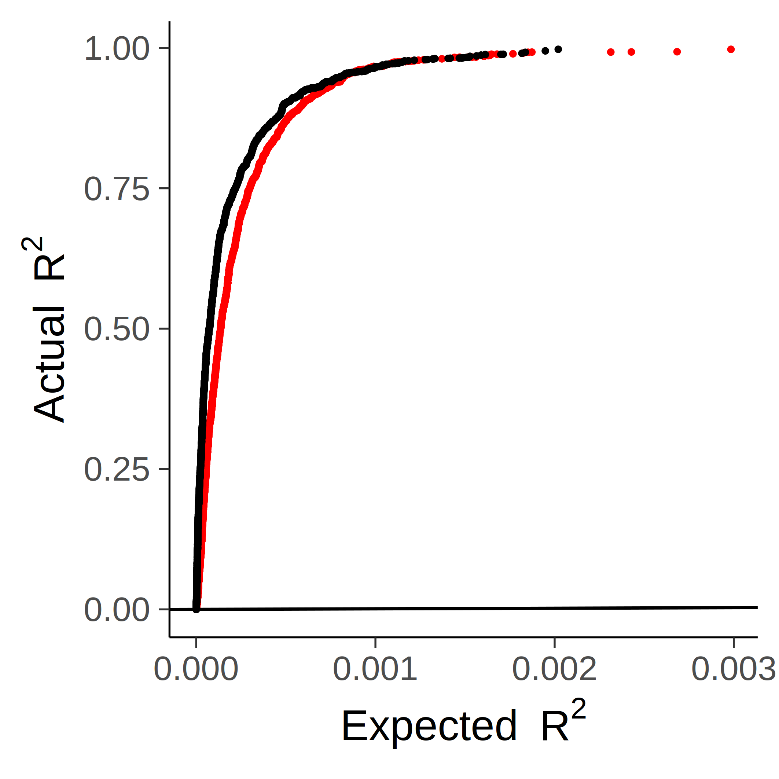


**S3 Fig**. **Correlation between imputed gene expression from independent predictive models of the same and different tissues.** The correlation R^2^ between imputed gene expression among ~5,000 unrelated CF GWAS patients, from CF LCL and GTEx_v7 LCL data sets (black) with 1,623 common genes, were plotted against expected R^2^ from null distribution (solid line almost flat at the bottom). Similar comparison between CF LCL and GTEx_v7 lung data sets (red), with 2,552 common genes are also plotted.


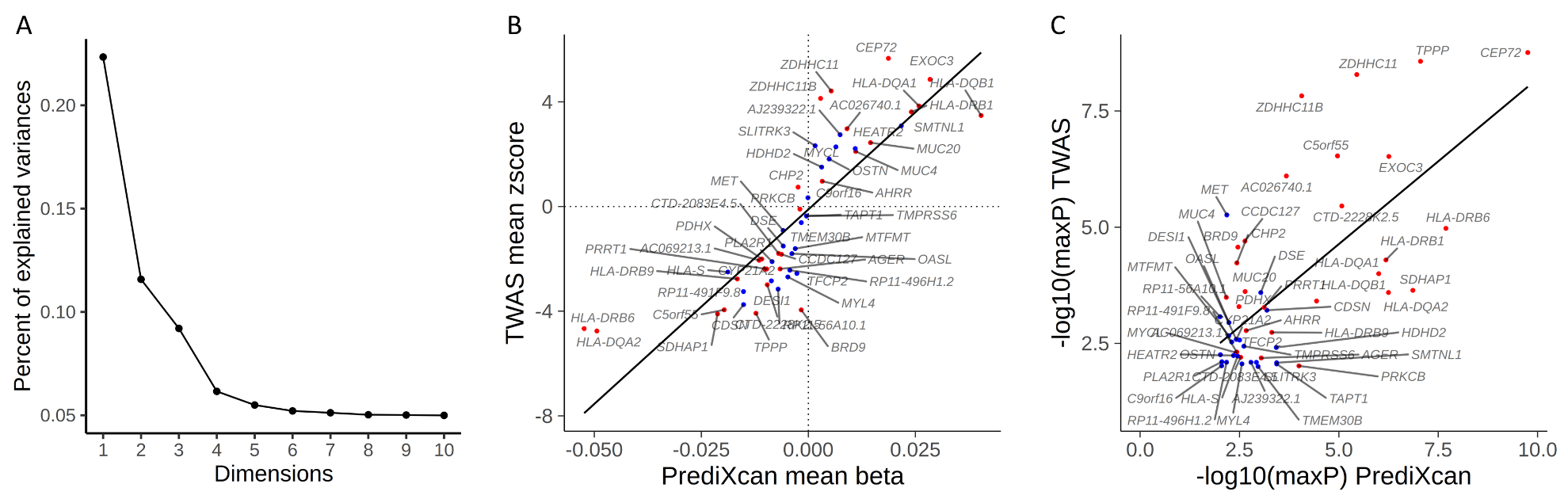


**S4 Fig**. **Correlation of CF lung disease association of imputed gene expression between PrediXcan and TWAS**. The percent of variances explained by the top 10 genotype principle components (PCs) were plotted in A, 4 PCs were used as covariates in phenotype association testing. The resultant mean effect sizes, calculated among multiple tissues, of the 52 consensus modifier genes from PrediXcan (using beta coefficients), and TWAS (using signed zscores) are plotted in B, while the maximal p-values between 2 meta-analyses for each platform are plotted in C. Red markers are genes near GWAS loci, and blue dots are novel modifier genes. Solid lines in B and C represent linear regression, while dotted lines in B mark the intercepts at zero.


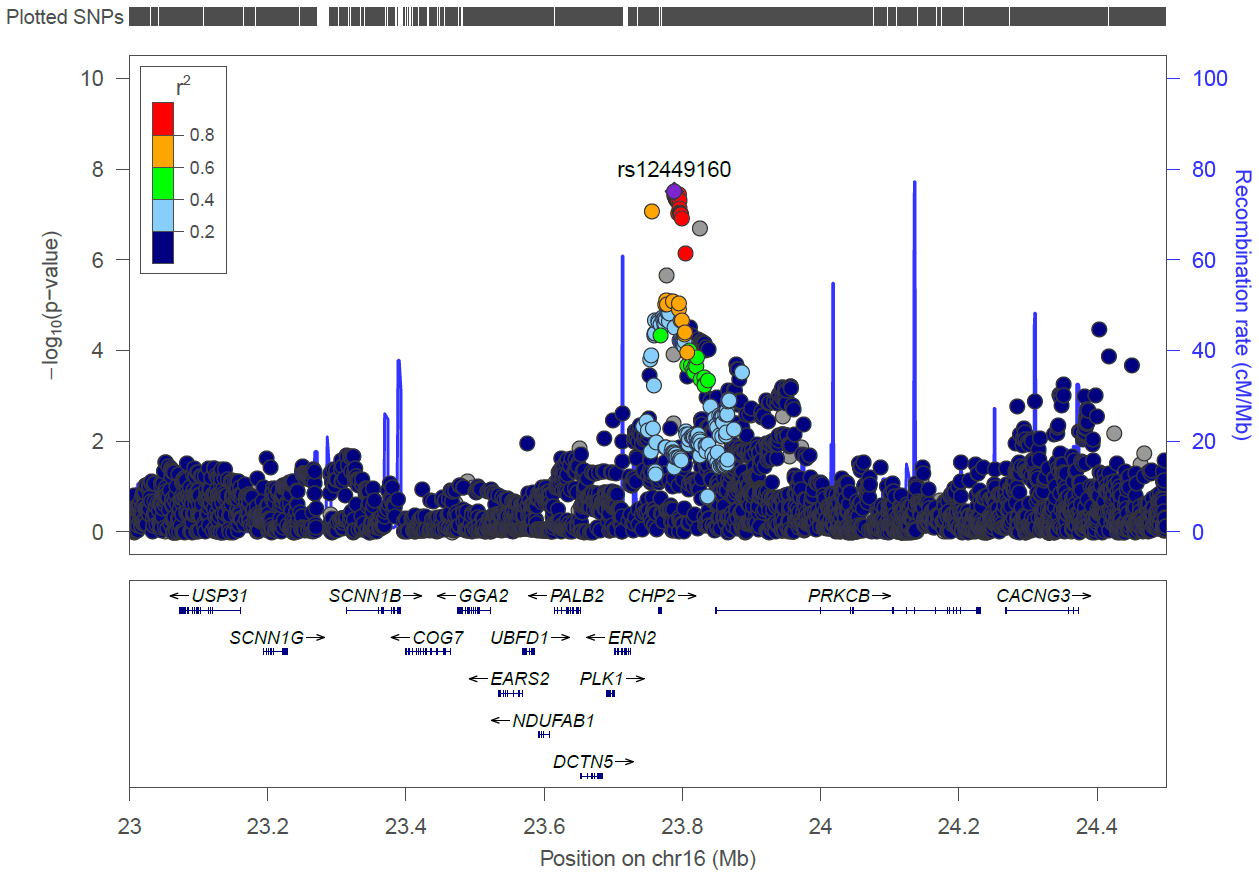


**S5 Fig**. **LocusZoom plot for the GWAS signal at chr16**. The association p-values from updated CF GWAS analysis based on latest imputation are plotted around the chr16 locus with LD *r*^2^ values derived from 1000 genomes EUR Nov 2014 release.


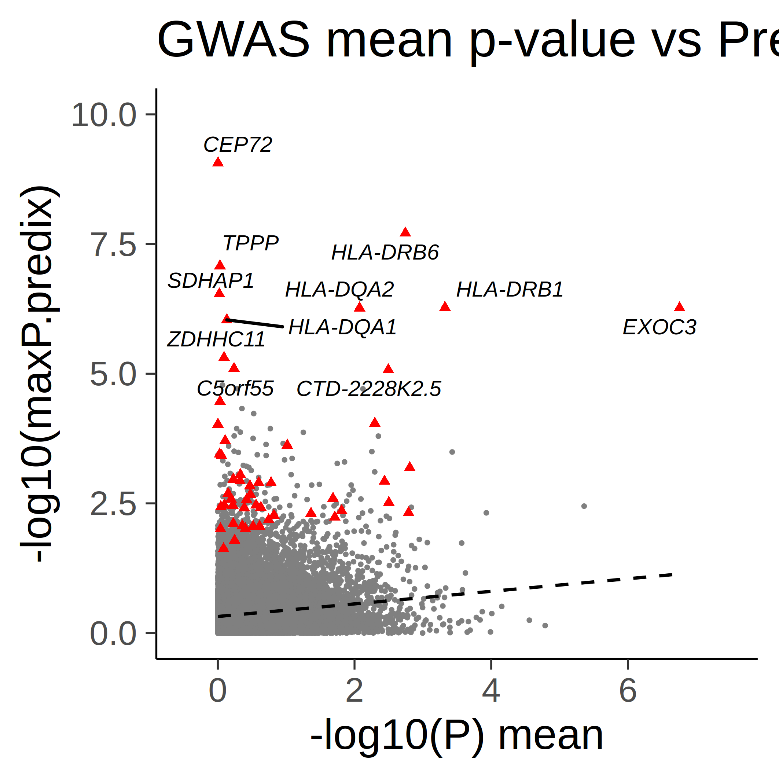


**S6 Fig**. **Correlation of CF lung disease association p-values between imputed gene expression and mean GWAS p-values.** The maximal association p-values (-log10 transformed) per gene from PrediXcan meta-analyses were plotted against the mean CF GWAS p-values among all SNPs in the predictive model for the same gene. The dashed line represents the linear regression line, while the top genes (p-value<10^-05^) from the 52 consensus modifier genes are highlighted with red triangles.


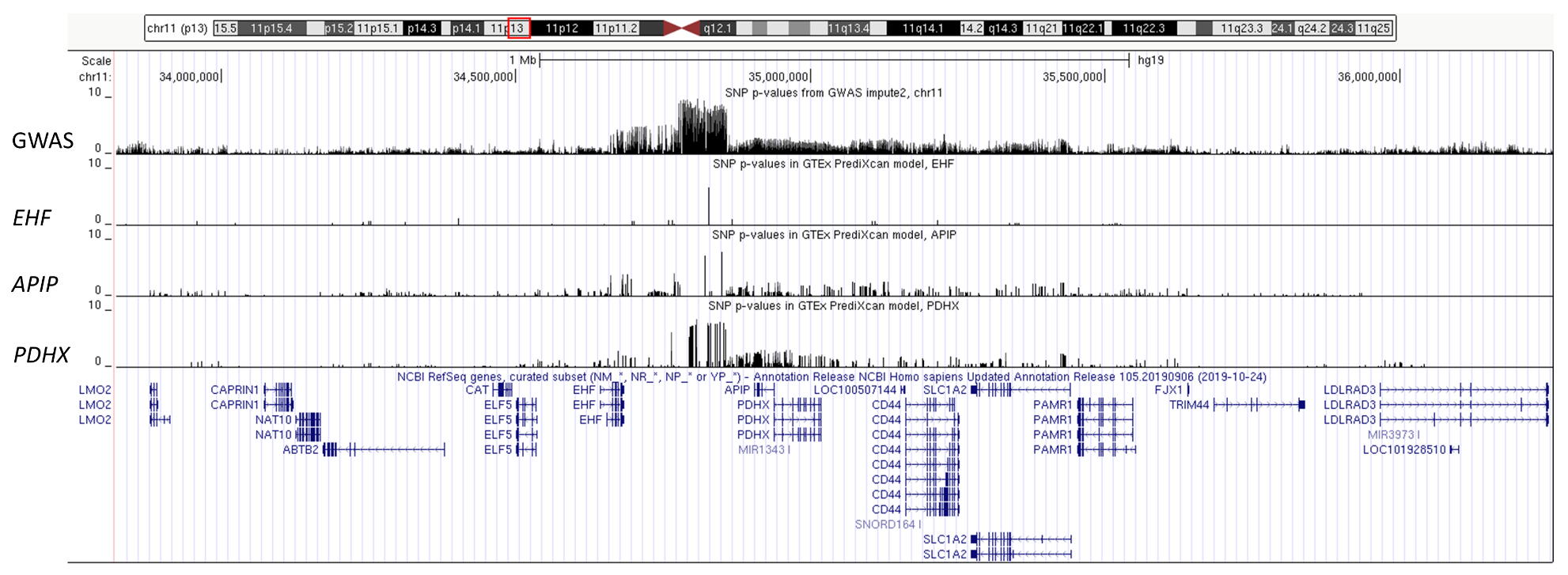


**S7 Fig**. **Comparison of predictive models SNPs at chr11 CF GWAS locus – full region**. The -log10 p-values from GWAS analysis were retrieved for *cis*-SNPs in PrediXcan predictive models from 48 GTEx tissues for *EHF*, *APIP*, and *PDHX*. These p-values were formatted as bedGraph files and displayed through the UCSC genome browser (http://genome.ucsc.edu/) as custom annotation tracks. The screenshot of the genome browser shows from top to bottom: GWAS results, SNPs in *EHF* gene expression imputation model, *APIP*, *PDHX*, and gene annotation from NCBI RefSeq genes.


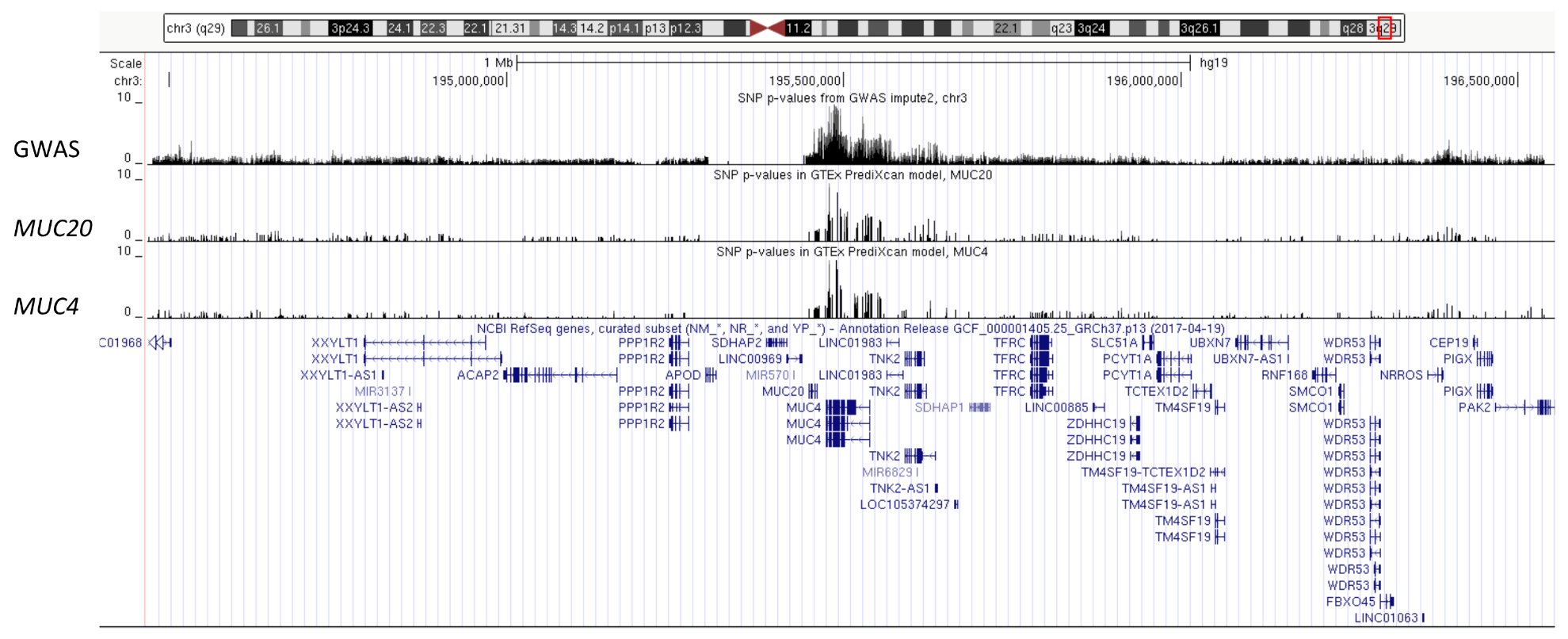


**S8 Fig**. **Comparison of predictive models SNPs at chr3 CF GWAS locus**. The -log10 p-values from GWAS analysis were retrieved for *cis*-SNPs in PrediXcan predictive models from 48 GTEx tissues for *MUC20*, and *MUC4*. These p-values were formatted as bedGraph files and displayed through the UCSC genome browser (http://genome.ucsc.edu/) as custom annotation tracks. The screenshot of the genome browser shows from top to bottom: GWAS results, SNPs in *MUC20* gene expression imputation model, those for *MUC4*, and gene annotation from GENCODE v19.

**
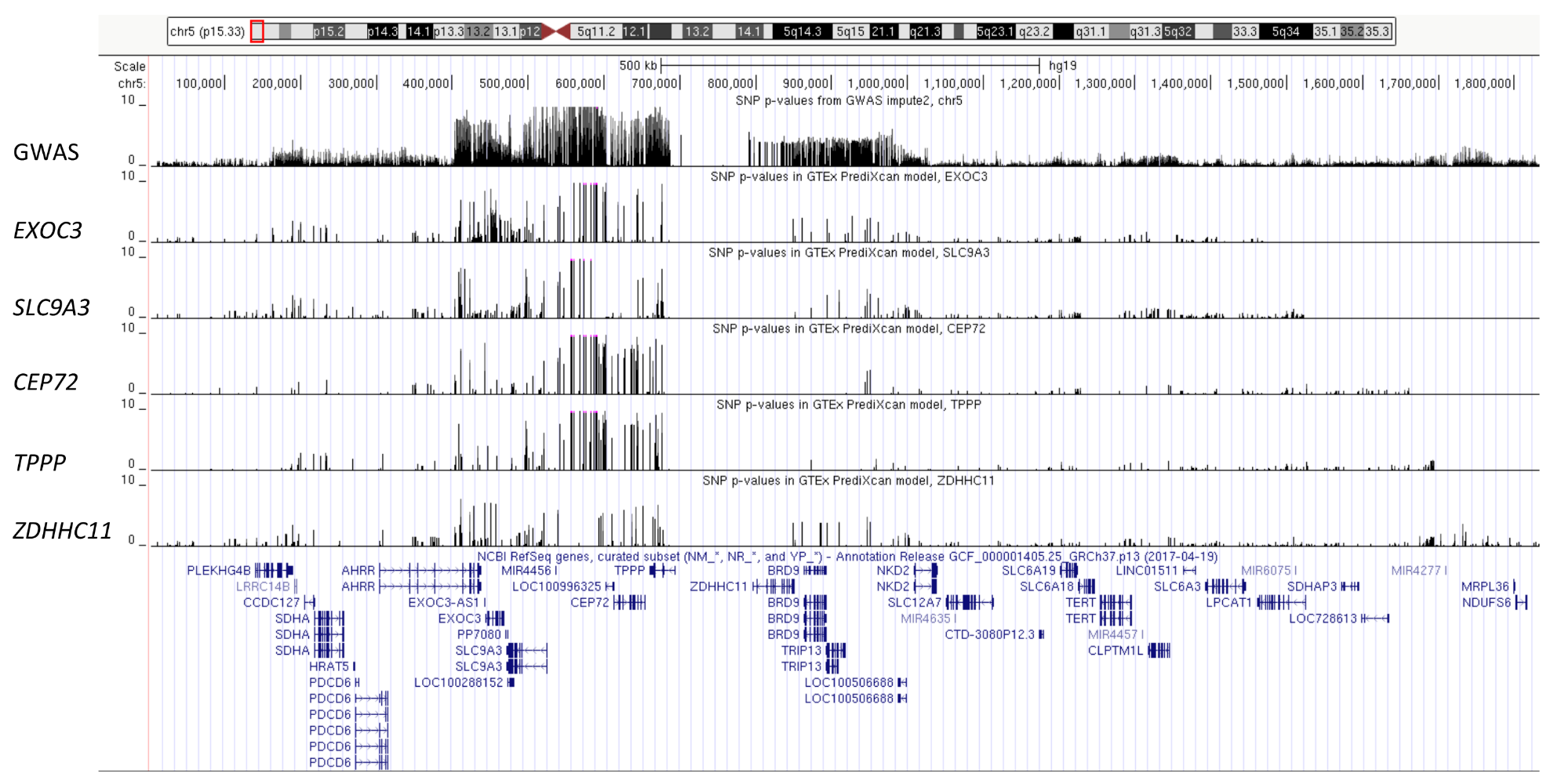
**

**S9 Fig**. **Comparison of predictive models SNPs at chr5 CF GWAS locus**. The -log10 p-values from GWAS analysis were retrieved for *cis*-SNPs in PrediXcan predictive models from 48 GTEx tissues for *CEP72*, *EXOC3*, *SLC9A3*, *TPPP*, and *ZDHHC11*. These p-values were formatted as bedGraph files and displayed through the UCSC genome browser (http://genome.ucsc.edu/) as custom annotation tracks. The screenshot of the genome browser shows from top to bottom: GWAS results, SNPs in *EXOC3* expression imputation model, those for *SLC9A3*, *CEP72*, *TPPP*, and *ZDHHC11*, and gene annotation from GENCODE v19.

**
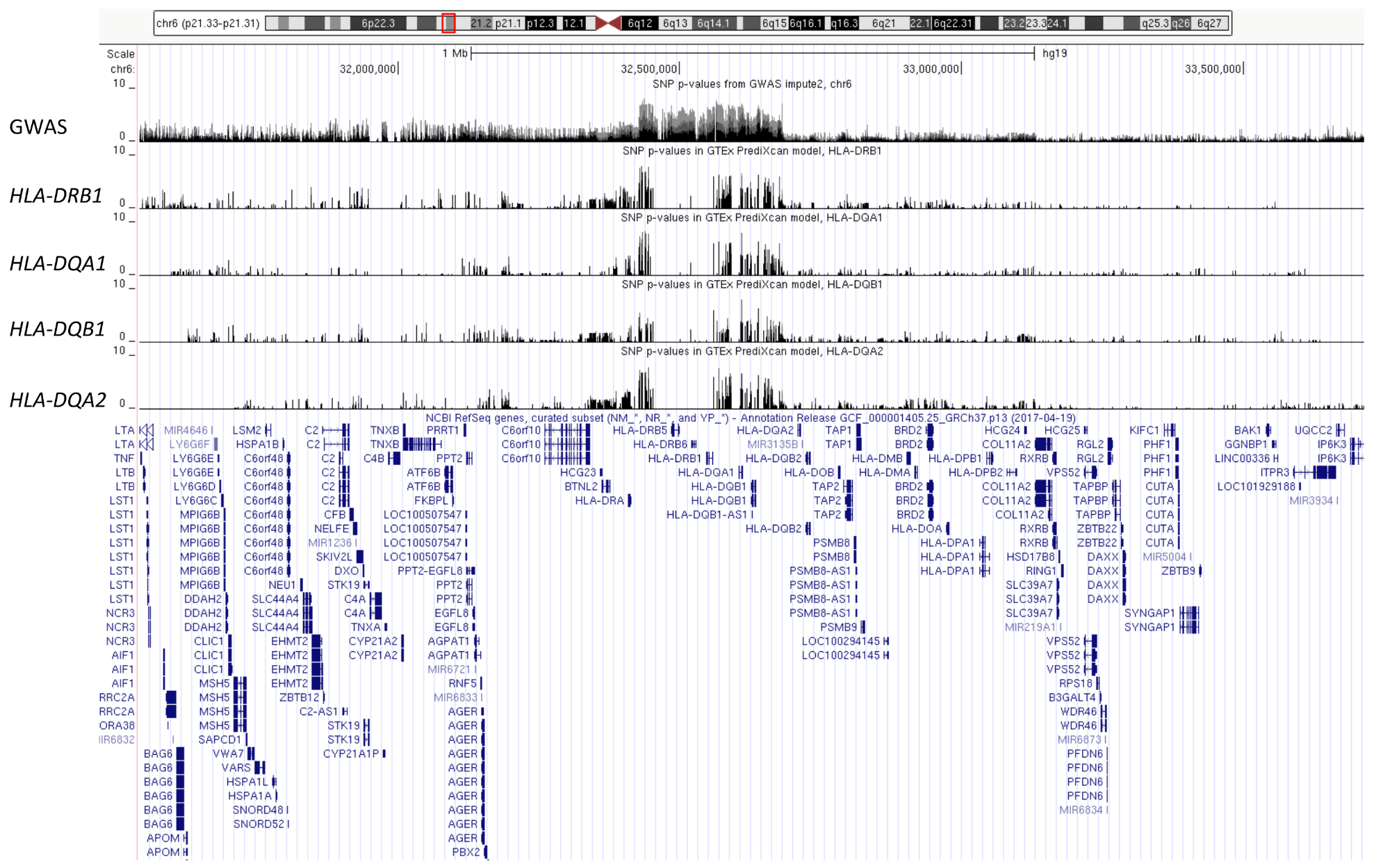
**

**S10 Fig**. **Comparison of predictive models SNPs at chr6 CF GWAS locus**. The -log10 p-values from GWAS analysis were retrieved for *cis*-SNPs in PrediXcan predictive models from 48 GTEx tissues for selected HLA class II genes. These p-values were formatted as bedGraph files and displayed through the UCSC genome browser (http://genome.ucsc.edu/) as custom annotation tracks. The screenshot of the genome browser shows from top to bottom: GWAS results, SNPs in *HLA-DRB1* gene expression imputation model, those for *HLA-DQA1*, *HLA-DQB1*, and *HLA-DQA2*, and gene annotation from NCBI RefSeq genes.

**
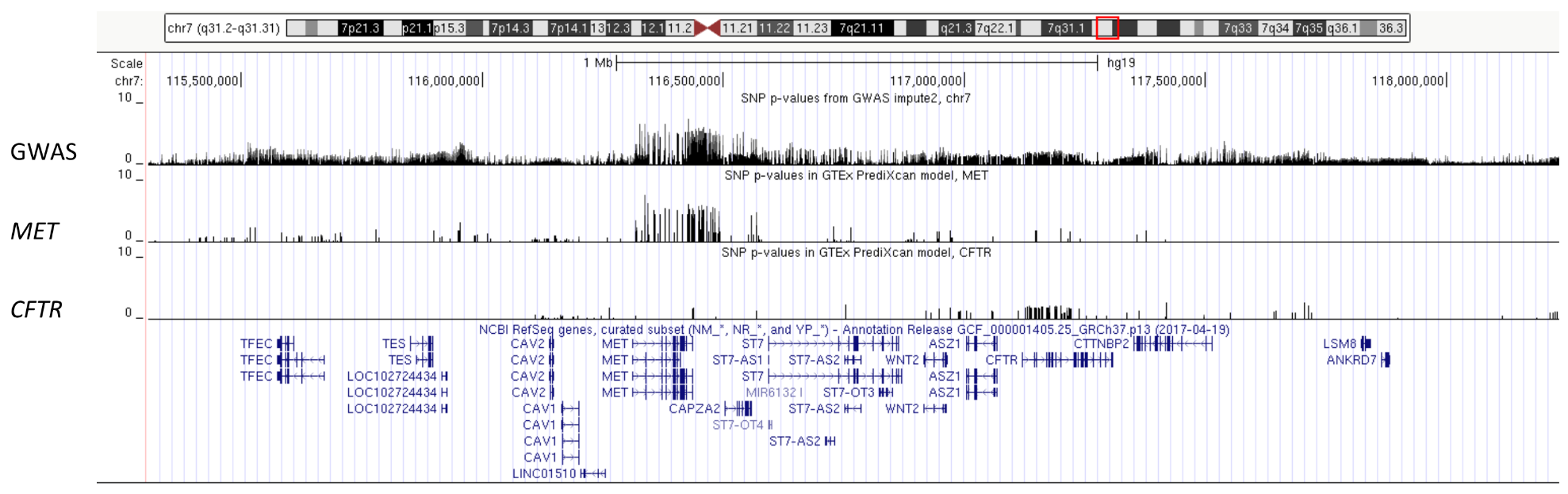
**

**S11 Fig**. **Comparison of predictive models SNPs at chr7 near *CFTR***. The -log10 p-values from GWAS analysis were retrieved for *cis*-SNPs in PrediXcan predictive models from 48 GTEx tissues for *MET*, and *CFTR*. These p-values were formatted as bedGraph files and displayed through the UCSC genome browser (http://genome.ucsc.edu/) as custom annotation tracks. The screenshot of the genome browser shows from top to bottom: GWAS results, SNPs in *MET* gene expression imputation model, those for *CFTR*, and gene annotation from GENCODE v19.

**
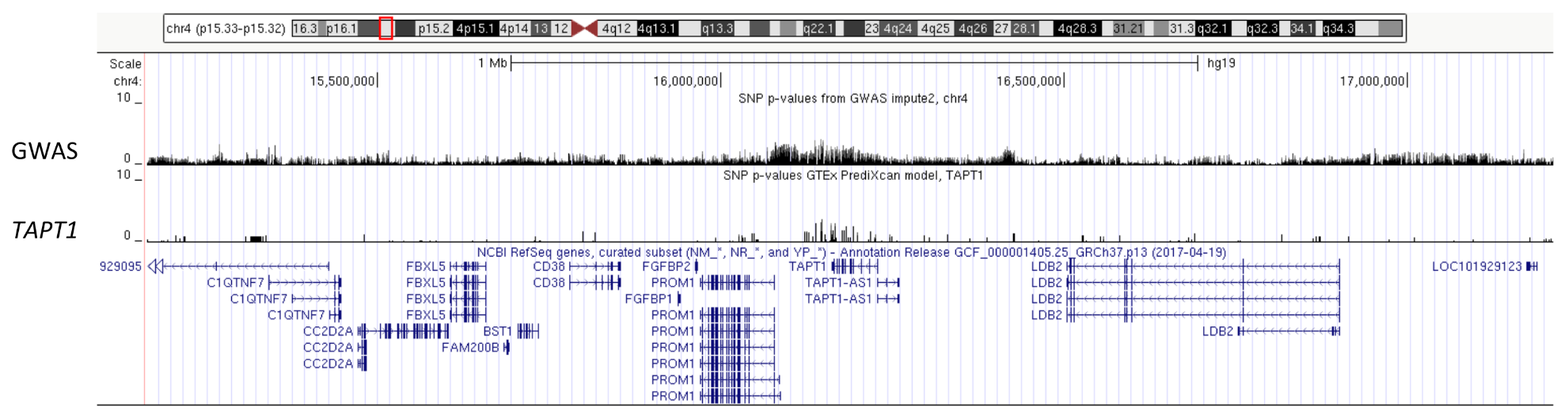
**

**S12 Fig**. **Comparison of predictive models SNPs at chr4**. The -log10 p-values from GWAS analysis were retrieved for *cis*-SNPs in PrediXcan predictive models from 48 GTEx tissues for *TAPT1*. These p-values were formatted as bedGraph files and displayed through the UCSC genome browser (http://genome.ucsc.edu/) as custom annotation tracks. The screenshot of the genome browser shows from top to bottom: GWAS results, SNPs in *TAPT1* gene expression imputation model, and gene annotation from GENCODE v19.


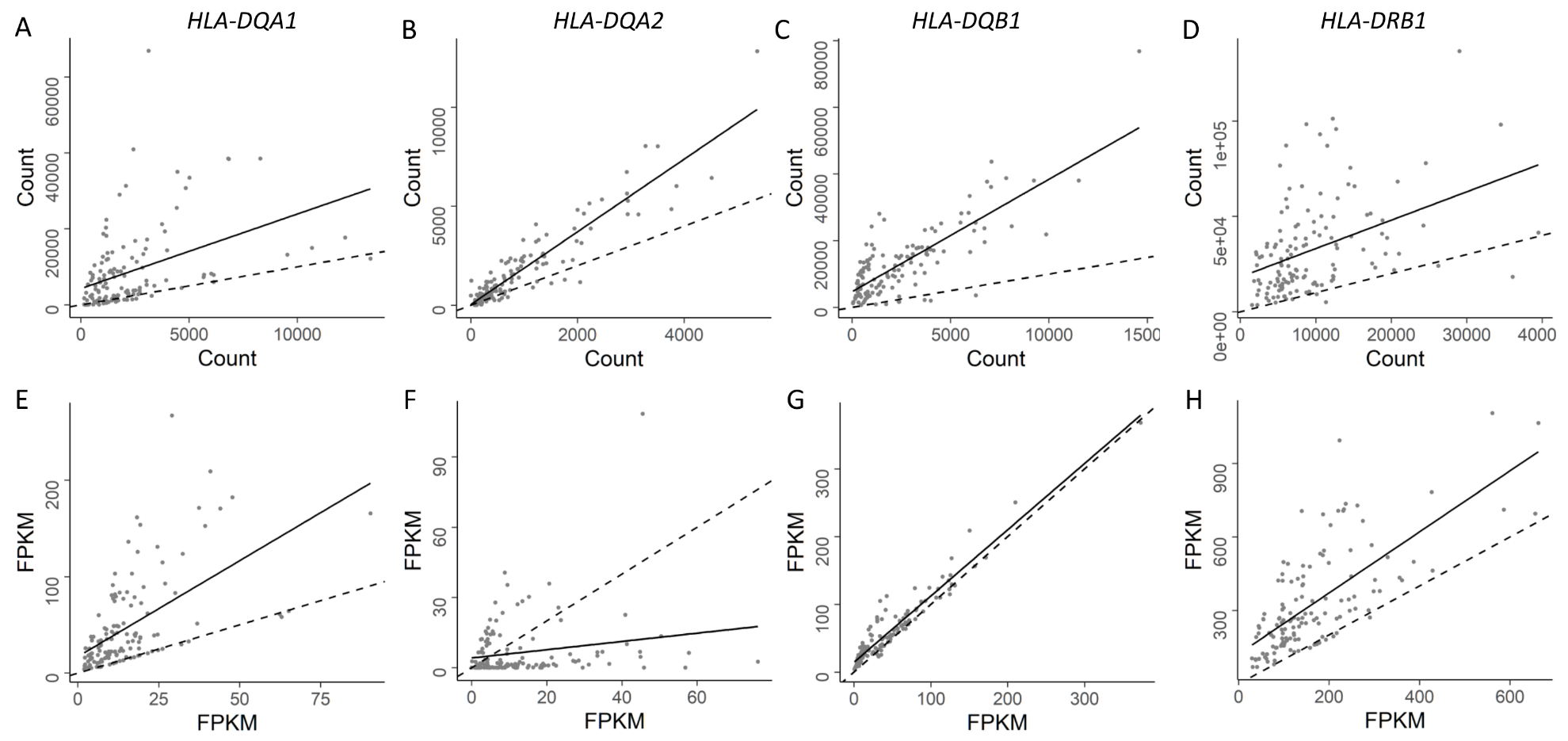


**S13 Fig**. **Comparison of gene expression quantification of selected HLA Class II genes between different protocols from CF nasal epithelial biopsy data set**. Four genes (*HLA-DQA1*, *HLA-DQA2*, *HLA-DQB1*, and *HLA-DRB1*) are shown from left to right, and top row (A, B, C, D) represents comparison of gene counts, while the bottom row (E, F, G, H) shows FPKM values. Dashed line denotes unity, while solid line represents linear regression line in each plot. For count data (top row), values from common reference genome mapping by HISAT2/StringTie (x-axis) are compared with AltHapAlignR (y-axis). The bottom row compares FPKM values from common reference genome mapping (x-axis), and alternative variant-aware all-inclusive assembly mapping (y-axis) protocol.

| **S1 Table.** **GSEA results from PrediXcan**  GSEA were performed with total number of imputed protein coding genes from multiple tissues pre-ranked by lung function association p-value.  **more extreme (*n*)** = number of times a better enrichment score was achieved over 1 million random permutations  **size** = number of protein coding genes in the pathway also present in our imputed expression dataset (n=16,431)  **Genes on the leading edge** = genes by rank responsible for increasing enrichment score (***bold***: consensus genes near GWAS loci; *underlined*: consensus genes outside GWAS loci) | | | | | |
| --- | --- | --- | --- | --- | --- |
| **Pathway ID** | **Description** | **p-value** | **more extreme (*n*)** | **size** | **Genes on the leading edge** |
| **Go Biological Processes** (http://geneontology.org/) | | | | | |
| GO:0060333 | interferon-gamma-mediated signaling pathway | 4.00E-06 | 3 | 65 | ***HLA-DQA2****,* ***HLA-DRB1****,* ***HLA-DQA1****, HLA-DQB2,* ***HLA-DQB1****, HLA-B, OASL, CD44, HLA-DRB5, CAMK2D, HLA-DRA, NCAM1, GBP1, HLA-C* |
| GO:0034629 | cellular protein-containing complex localization | 7.92E-05 | 78 | 21 | ***CEP72****,* ***EXOC3****, NDC1, MIOS* |
| GO:1904779 | regulation of protein localization to centrosome | 1.96E-04 | 189 | 10 | ***CEP72****, MARK4* |
| GO:0033227 | dsRNA transport | 4.90E-04 | 58 | 5 | *RFTN1, FLOT1, RFTN2, SIDT1, SIDT2* |
| GO:0034383 | low-density lipoprotein particle clearance | 5.28E-04 | 527 | 24 | *SOAT1, LDLRAP1, HMOX1, AP2S1, DGAT2, APOB* |
| GO:0035635 | entry of bacterium into host cell | 7.30E-04 | 691 | 8 | *MET, CBLL1, CTNND1, CTNNB1* |
| GO:0002768 | immune response-regulating cell surface receptor signaling pathway | 1.70E-03 | 1696 | 307 | ***HLA-DQA2****,* ***HLA-DRB1****,* ***HLA-DQA1****, HLA-DQB2,* ***HLA-DQB1****,* ***PRKCB****, BTNL2,* ***MUC20****, PIK3R2, SPG21,* ***MUC4****, VAV3, ICAM3, CALM1, HLA-DRB5, STOML2, FCER1A, GATA3, KIT, HLA-DRA, MEF2C, BTN3A2, EP300, PSMB4, PLEKHA1, THEMIS, PSMD14, PSMB9, CYFIP2, PSMC2, MUC1, SKP1, LILRA4, MYO10, PSMB2, PSMF1, FCGR2B, PIGR, WIPF2, PSMD9, LILRA2, BCL2, NFKB1, THY1, PSMB1, KRAS, CDC42, PAG1, SYK, MAP2K7, PSMB8, BTN3A3, PTPN6, KIR2DL1, RC3H2, PSME1, LYN, HCK, CD79A, CLEC6A, ARPC1B, GPR32, MUC5B, VTCN1, THEMIS2, PSEN1, MAPK3, NCR3, FPR2, MUC16, BTRC, CARD11, MUC21, FGR, DENND1B, PRKCE, NFKBID, YES1, PSMD8, PDE4B, FCGR2A, MUC3A, BTLA, PPP3CA, PSMA6, PSMA7, ICOSLG, HLA-DPB1, SLA2* |
| GO:0042461 | photoreceptor cell development | 1.73E-03 | 1731 | 31 | *THRB, ARL3, RP1, CCDC66, CRB1, TH, GNAT2, RP1L1, RPGRIP1, THY1, RORB, NTRK2, GNGT1* |
| GO:0019886 | antigen processing and presentation of exogenous peptide antigen via MHC class II | 2.23E-03 | 2230 | 89 | ***HLA-DQA2****,* ***HLA-DRB1****,* ***HLA-DQA1****, HLA-DQB2,* ***HLA-DQB1****, AP1S1, HLA-DRB5, AP2S1, HLA-DRA, KIF2A, HLA-DMB, HLA-DMA, CTSF, DCTN3, FCGR2B, CAPZA1, DYNC1H1, KIF3C, KIF15, DYNLL1, DCTN4, CLTA, KLC1, CTSS, AP1M2* |
| GO:0002891 | positive regulation of immunoglobulin mediated immune response | 2.24E-03 | 2242 | 32 | *TFRC, ATAD5, XCL1, TNF, RIF1, CLCF1, TNFSF13, TGFB1, LTA, EXOSC3, FCER2, TNFSF4, STAT6, MLH1, C3, HLA-E* |
| GO:0048012 | hepatocyte growth factor receptor signaling pathway | 2.41E-03 | 2364 | 12 | ***MUC20****, STMN1, MET, HGF* |
| GO:0042407 | cristae formation | 2.58E-03 | 2403 | 7 | *CHCHD3, SAMM50* |
| GO:0050851 | antigen receptor-mediated signaling pathway | 2.71E-03 | 2706 | 174 | ***HLA-DQA2****,* ***HLA-DRB1****,* ***HLA-DQA1****, HLA-DQB2,* ***HLA-DQB1****,* ***PRKCB****, BTNL2, PIK3R2, SPG21, VAV3, HLA-DRB5, STOML2, GATA3, HLA-DRA, MEF2C, BTN3A2, PSMB4, PLEKHA1, THEMIS, PSMD14, PSMB9, PSMC2, SKP1, PSMB2, PSMF1, PSMD9, BCL2, NFKB1, THY1, PSMB1, PAG1, SYK, PSMB8, BTN3A3, PTPN6, RC3H2, PSME1, LYN, CD79A, VTCN1, THEMIS2, PSEN1, BTRC, CARD11, DENND1B, NFKBID, PSMD8, PDE4B, PSMA6, PSMA7, ICOSLG, HLA-DPB1, SLA2, CRKL, PAK2, BTN2A1* |
| GO:0002495 | antigen processing and presentation of peptide antigen via MHC class II | 2.72E-03 | 2724 | 90 | ***HLA-DQA2****,* ***HLA-DRB1****,* ***HLA-DQA1****, HLA-DQB2,* ***HLA-DQB1****, AP1S1, HLA-DRB5, AP2S1, HLA-DRA, KIF2A, HLA-DMB, HLA-DMA, CTSF, DCTN3, FCGR2B, CAPZA1, DYNC1H1, KIF3C, KIF15, DYNLL1, DCTN4, CLTA, KLC1, CTSS, AP1M2* |
| GO:0002504 | antigen processing and presentation of peptide or polysaccharide antigen via MHC class II | 2.72E-03 | 2724 | 90 | ***HLA-DQA2****,* ***HLA-DRB1****,* ***HLA-DQA1****, HLA-DQB2,* ***HLA-DQB1****, AP1S1, HLA-DRB5, AP2S1, HLA-DRA, KIF2A, HLA-DMB, HLA-DMA, CTSF, DCTN3, FCGR2B, CAPZA1, DYNC1H1, KIF3C, KIF15, DYNLL1, DCTN4, CLTA, KLC1, CTSS, AP1M2* |
| GO:0045191 | regulation of isotype switching | 2.95E-03 | 2953 | 29 | *TFRC, ATAD5, RIF1, CLCF1, TNFSF13, IL27RA, TGFB1, EXOSC3, APLF, SUPT6H, TNFSF4, STAT6, MLH1* |
| GO:0002429 | immune response-activating cell surface receptor signaling pathway | 3.35E-03 | 3353 | 275 | ***HLA-DQA2****,* ***HLA-DRB1****,* ***HLA-DQA1****, HLA-DQB2,* ***HLA-DQB1****,* ***PRKCB****, BTNL2,* ***MUC20****, PIK3R2, SPG21,* ***MUC4****, VAV3, ICAM3, HLA-DRB5, STOML2, GATA3, HLA-DRA, MEF2C, BTN3A2, EP300, PSMB4, PLEKHA1, THEMIS, PSMD14, PSMB9, CYFIP2, PSMC2, MUC1, SKP1, MYO10, PSMB2, PSMF1, FCGR2B, WIPF2, PSMD9, LILRA2, BCL2, NFKB1, THY1, PSMB1, KRAS, CDC42, PAG1, SYK, PSMB8, BTN3A3, PTPN6, RC3H2, PSME1, LYN, HCK, CD79A, CLEC6A, ARPC1B, GPR32, MUC5B, VTCN1, THEMIS2, PSEN1, MAPK3, NCR3, FPR2, MUC16, BTRC, CARD11, MUC21, FGR, DENND1B, PRKCE, NFKBID, YES1, PSMD8, PDE4B, FCGR2A, MUC3A* |
| GO:0045830 | positive regulation of isotype switching | 3.70E-03 | 3696 | 22 | *TFRC, ATAD5, RIF1, CLCF1, TNFSF13, TGFB1, EXOSC3, TNFSF4, STAT6, MLH1* |
| GO:0002253 | activation of immune response | 3.97E-03 | 3966 | 432 | ***HLA-DQA2****,* ***HLA-DRB1****,* ***HLA-DQA1****, HLA-DQB2,* ***HLA-DQB1****,* ***PRKCB****, C2, BTNL2,* ***MUC20****, PIK3R2, SPG21,* ***MUC4****, VAV3, TREX1, ICAM3, PIK3AP1, LRRC19, HLA-DRB5, CFHR1, STOML2, APOB, GATA3, S100A1, XRCC6, HLA-DRA, PGLYRP3, MEF2C, MASP1, XRCC5, S100A14, BTN3A2, EP300, PSMB4, PLEKHA1, THEMIS, PSMD14, PSMB9, CYFIP2, C4B, C4A, PSMC2, MUC1, SKP1, TLR3, CFH, MARCO, MYO10, AIM2, TLR2, PSMB2, RGCC, MYD88, PSMF1, FCGR2B, WIPF2, PSMD9, LILRA2, BCL2, NFKB1, THY1, TANK, PSMB1, KRAS, CDC42, C1R, PAG1, SYK, CLEC7A, PSMB8, BTN3A3, CTSK, PTPN6, HEXIM1, LY96, RC3H2, CFB, CNPY3, PSME1, RBM14, TBK1, IRF7, LYN, HCK, CTSS, CD79A, DDX58, CLEC6A, ARPC1B, GPR32, MUC5B, SFTPA1, VTCN1, THEMIS2, PSEN1, MAPK3, NCR3, C3, FPR2, FADD, MUC16, BTRC, CARD11, MASP2, SCARA3, MUC21, FGR, DENND1B, PRKCE, NFKBID, YES1, PSMD8, PDE4B, FCGR2A, PIK3C3, MUC3A* |
| GO:0007099 | centriole replication | 4.88E-03 | 4827 | 14 | ***CEP72****, CEP135, WDR62, RTTN, CEP152, PLK4* |
| GO:0006304 | DNA modification | 5.18E-03 | 5184 | 77 | *TDRKH, MOV10L1, TREX1, MUTYH, MTA2, ALKBH5, DNMT3B, MBD4, APOBEC3H, TRIM28, APOBEC3F, TDRD5, TET3, PARP2, EXOSC3, EHMT2, PIWIL4, TET2, GNAS* |
| GO:0075713 | establishment of integrated proviral latency | 5.41E-03 | 5128 | 8 | *LIG4, XRCC6, XRCC5, HMGA1, XRCC4* |
| GO:0045652 | regulation of megakaryocyte differentiation | 5.47E-03 | 5468 | 44 | *SCIN, HMGB2, THPO, AGO4, TNRC6A, MEF2C, EP300, KMT2C, PRMT1, AGO1, L3MBTL1, RUNX1, EIF6, CBFB, RBM15, MYL9, ITGA2B, TESC, HIST1H3A, MOV10, LOX* |
| GO:0007614 | short-term memory | 5.54E-03 | 5320 | 9 | *CUX2, ADNP, CHRNA7, COMT* |
| GO:0043586 | tongue development | 6.09E-03 | 5905 | 10 | *NKX2-6, KIT, LEF1, SIX4* |
| GO:0019915 | lipid storage | 6.27E-03 | 6267 | 26 | *SOAT1, BSCL2, NRIP1, TNF, DGAT2* |
| GO:0042462 | eye photoreceptor cell development | 6.28E-03 | 6278 | 29 | *THRB, RP1, CCDC66, CRB1, TH, GNAT2, RPGRIP1, THY1, RORB, NTRK2, GNGT1* |
| GO:0000018 | regulation of DNA recombination | 6.41E-03 | 6406 | 77 | *TFRC, ATAD5, RIF1, UBE2B, CLCF1, KPNA1, TNFSF13, FIGNL1, TERF2IP, PPP4R2, IL27RA, TGFB1, MMS19, H1FOO, ZRANB3, EXOSC3, RPA2, APLF, SMARCAD1, SUPT6H, TNFSF4, PPP4C, STAT6, MLH1, PARPBP, RECQL5* |
| GO:0071599 | otic vesicle development | 6.58E-03 | 5784 | 5 | *SIX1, GATA3, COL2A1* |
| GO:0002764 | immune response-regulating signaling pathway | 7.13E-03 | 7130 | 408 | ***HLA-DQA2****,* ***HLA-DRB1****,* ***HLA-DQA1****, HLA-DQB2,* ***HLA-DQB1****,* ***PRKCB****, BTNL2,* ***MUC20****, PIK3R2, SPG21,* ***MUC4****, VAV3, ICAM3, PIK3AP1, CALM1, LRRC19, HLA-DRB5, STOML2, FCER1A, APOB, GATA3, KIT, S100A1, HLA-DRA, PGLYRP3, MEF2C, S100A14, BTN3A2, EP300, PSMB4, PLEKHA1, THEMIS, PSMD14, PSMB9, CYFIP2, PSMC2, MUC1, SKP1, TLR3, MARCO, LILRA4, MYO10, TLR2, PSMB2, MYD88, PSMF1, FCGR2B, PIGR, WIPF2, PSMD9, LILRA2, BCL2, NFKB1, THY1, TANK, PSMB1, KRAS, CDC42, PAG1, SYK, MAP2K7, CLEC7A, PSMB8, BTN3A3, CTSK, PTPN6, KIR2DL1, LY96, RC3H2, CNPY3, PSME1, TBK1, IRF7, LYN, HCK, CTSS, CD79A, DDX58, CLEC6A, ARPC1B, GPR32, MUC5B, SFTPA1, VTCN1, THEMIS2, PSEN1, MAPK3, NCR3, FPR2, FADD, MUC16, BTRC, CARD11, SCARA3, MUC21, FGR, DENND1B, PRKCE, NFKBID, YES1, PSMD8, PDE4B, FCGR2A, PIK3C3, MUC3A, BTLA, PPP3CA, PSMA6, PSMA7, ICOSLG, HLA-DPB1, SLA2* |
| GO:0031503 | protein-containing complex localization | 7.18E-03 | 7177 | 124 | ***CEP72****,* ***EXOC3****, NDC1, IFT74, ICK, ARL3, IFT88, WDR34, MIOS, ARHGAP44, DNM1, EPS15, ABCE1, IFT81, RPS15, IFT80, IFT122, TRAF3IP1, TNPO1, RRS1, CALY, EIF6, LCA5, CLSTN1, SHISA6, KIF3C, RAN, SDAD1, HSPB11, MZT1, DYNLL1* |
| GO:0030890 | positive regulation of B cell proliferation | 7.61E-03 | 7608 | 34 | *TFRC, VAV3, ATAD5, TNFRSF13C, CLCF1, MEF2C, CHRNB2, BCL2, CD81* |
| GO:0009582 | detection of abiotic stimulus | 7.68E-03 | 7677 | 97 | *SERPINE2, RP1, CCDC66, CALCA, ANO1, KIT, TACR1, TAC1, MMP24, GRK1, PDC, ASIC2, GRM6, BEST1, GNAT2, DRGX, OPN1SW, ATP8A2, TMC1, CXCL12, RGS9BP, JUP, PITPNM1, GNGT1, TRPV1, KCNA1, GPR52, RPE65, OPN5, PKD2L2, ANO3, GNA11, CHRNA10, MKKS, EPHB1, TRPM8, PLEKHB1* |
| GO:0010757 | negative regulation of plasminogen activation | 7.70E-03 | 6776 | 5 | *SERPINE2, THBS1* |
| GO:0045091 | regulation of single stranded viral RNA replication via double stranded DNA intermediate | 7.72E-03 | 7651 | 15 | *FAM208A, APOBEC3H, TRIM28, SETDB1, APOBEC3F, MORC2* |
| GO:0034381 | plasma lipoprotein particle clearance | 7.76E-03 | 7758 | 39 | *SOAT1, LDLRAP1, APOC2, HMOX1, AP2S1, DGAT2, APOB, APOM, EHD1, CLTA, HDLBP, APOC1, APOA1, APOC4, APOA2, APOE* |
| GO:0006505 | GPI anchor metabolic process | 7.80E-03 | 7792 | 23 | *PIGW, PIGO, PIGP, PYURF, PIGH, PGAP1, PGAP3, DPM1* |
| GO:0042535 | positive regulation of tumor necrosis factor biosynthetic process | 7.83E-03 | 7640 | 11 | ***AGER****, AZU1* |
| GO:0002757 | immune response-activating signal transduction | 7.91E-03 | 7912 | 374 | ***HLA-DQA2****,* ***HLA-DRB1****,* ***HLA-DQA1****, HLA-DQB2,* ***HLA-DQB1****,* ***PRKCB****, BTNL2,* ***MUC20****, PIK3R2, SPG21,* ***MUC4****, VAV3, ICAM3, PIK3AP1, LRRC19, HLA-DRB5, STOML2, APOB, GATA3, S100A1, HLA-DRA, PGLYRP3, MEF2C, S100A14, BTN3A2, EP300, PSMB4, PLEKHA1, THEMIS, PSMD14, PSMB9, CYFIP2, PSMC2, MUC1, SKP1, TLR3, MARCO, MYO10, TLR2, PSMB2, MYD88, PSMF1, FCGR2B, WIPF2, PSMD9, LILRA2, BCL2, NFKB1, THY1, TANK, PSMB1, KRAS, CDC42, PAG1, SYK, CLEC7A, PSMB8, BTN3A3, CTSK, PTPN6, LY96, RC3H2, CNPY3, PSME1, TBK1, IRF7, LYN, HCK, CTSS, CD79A, DDX58, CLEC6A, ARPC1B, GPR32, MUC5B, SFTPA1, VTCN1, THEMIS2, PSEN1, MAPK3, NCR3, FPR2, FADD, MUC16, BTRC, CARD11, SCARA3, MUC21, FGR, DENND1B, PRKCE, NFKBID, YES1, PSMD8, PDE4B, FCGR2A, PIK3C3, MUC3A* |
| GO:0043923 | positive regulation by host of viral transcription | 8.08E-03 | 8040 | 17 | *TFAP4, LEF1, CTDP1, EP300, TAF11, SMARCA4* |
| GO:0010829 | negative regulation of glucose transmembrane transport | 8.18E-03 | 8084 | 14 | ***PRKCB****, OSTN, TNF, APPL2* |
| GO:0018230 | peptidyl-L-cysteine S-palmitoylation | 8.52E-03 | 8492 | 19 | ***ZDHHC11****,* ***ZDHHC11B****, ZDHHC19* |
| GO:0035518 | histone H2A monoubiquitination | 8.63E-03 | 8532 | 14 | *DDB1, BMI1, SKP1, KDM2B, RING1, RYBP, PCGF6* |
| GO:0048002 | antigen processing and presentation of peptide antigen | 9.20E-03 | 9202 | 167 | ***HLA-DQA2****,* ***HLA-DRB1****,* ***HLA-DQA1****, HLA-DQB2,* ***HLA-DQB1****, HLA-B, AP1S1, HLA-DRB5, AP2S1, HLA-DRA, CD207, PSMB4, KIF2A, LNPEP, PSMD14, PSMB9, HLA-DMB, HLA-C, PSMC2, HLA-DMA, CTSF, PSMB2, DCTN3, PSMF1, FCGR2B, PSMD9, CAPZA1, DYNC1H1, PSMB1, NCF1, PSMB8, KIF3C, KIF15, DYNLL1, PSME1, DCTN4, CLTA, VAMP3, KLC1, CTSS, AP1M2, KIF23, KIF4B, HLA-E, PSMD8, KIF5A, CAPZA2, OSBPL1A, PSMA6, PSMA7, HLA-DPB1, TAP1, KIF3B, SEC24D* |
| GO:0033522 | histone H2A ubiquitination | 9.34E-03 | 9303 | 18 | *UBE2B, DDB1, BMI1, UBR2, SKP1, KDM2B, RING1, RYBP, PCGF6* |
| GO:0042953 | lipoprotein transport | 9.74E-03 | 9635 | 14 | ***PRKCB****, APOC2, APOB* |
| GO:2001033 | negative regulation of double-strand break repair via nonhomologous end joining | 9.89E-03 | 8700 | 5 | *AUNIP, TFIP11* |
|  |  |  |  |  |  |
| **Biosystems** (https://www.ncbi.nlm.nih.gov/biosystems/) | | | | | |
| REACTOME_R_HSA_202433 | Generation of second messenger molecules | 3.00E-06 | 2 | 27 | ***HLA-DQA2****,* ***HLA-DRB1****,* ***HLA-DQA1****, HLA-DQB2,* ***HLA-DQB1****, HLA-DRB5, HLA-DRA* |
| KEGG_hsa05310 | Asthma | 3.00E-06 | 2 | 25 | ***HLA-DQA2****,* ***HLA-DRB1****,* ***HLA-DQA1****,* ***HLA-DQB1****, PRG2, TNF, HLA-DRB5, FCER1A, HLA-DRA, HLA-DMB, HLA-DMA* |
| REACTOME_R_HSA_202430 | Translocation of ZAP-70 to Immunological synapse | 3.03E-06 | 2 | 15 | ***HLA-DQA2****,* ***HLA-DRB1****,* ***HLA-DQA1****, HLA-DQB2,* ***HLA-DQB1****, HLA-DRB5, HLA-DRA* |
| REACTOME_R_HSA_202427 | Phosphorylation of CD3 and TCR zeta chains | 6.02E-06 | 5 | 18 | ***HLA-DQA2****,* ***HLA-DRB1****,* ***HLA-DQA1****, HLA-DQB2,* ***HLA-DQB1****, HLA-DRB5, HLA-DRA* |
| KEGG_hsa05416 | Viral myocarditis | 8.00E-06 | 7 | 53 | ***HLA-DQA2****,* ***HLA-DRB1****,* ***HLA-DQA1****,* ***HLA-DQB1****, HLA-B, RAC2, HLA-DRB5, HLA-DRA, HLA-DMB, HLA-C, HLA-DMA* |
| KEGG_hsa04612 | Antigen processing and presentation | 1.10E-05 | 10 | 62 | ***HLA-DQA2****,* ***HLA-DRB1****,* ***HLA-DQA1****,* ***HLA-DQB1****, HLA-B, HSPA1L, TNF, HLA-DRB5, HSPA1B, KLRD1, HLA-DRA, HLA-DMB, HLA-C, HLA-DMA* |
| REACTOME_R_HSA_389948 | PD-1 signaling | 1.31E-05 | 12 | 18 | ***HLA-DQA2****,* ***HLA-DRB1****,* ***HLA-DQA1****, HLA-DQB2,* ***HLA-DQB1****, HLA-DRB5, HLA-DRA* |
| KEGG_hsa05332 | Graft-versus-host disease | 1.70E-05 | 16 | 35 | ***HLA-DQA2****,* ***HLA-DRB1****,* ***HLA-DQA1****,* ***HLA-DQB1****, HLA-B, TNF, HLA-DRB5, KLRD1, HLA-DRA, HLA-DMB, HLA-C, HLA-DMA* |
| KEGG_hsa05330 | Allograft rejection | 1.80E-05 | 17 | 31 | ***HLA-DQA2****,* ***HLA-DRB1****,* ***HLA-DQA1****,* ***HLA-DQB1****, HLA-B, TNF, HLA-DRB5, HLA-DRA, HLA-DMB, HLA-C, HLA-DMA* |
| REACTOME_R_HSA_388841 | Costimulation by the CD28 family | 2.00E-05 | 19 | 61 | ***HLA-DQA2****,* ***HLA-DRB1****,* ***HLA-DQA1****, HLA-DQB2,* ***HLA-DQB1****, PIK3R2, PIK3R3, HLA-DRB5, MAP3K8, HLA-DRA* |
| KEGG_hsa05320 | Autoimmune thyroid disease | 2.20E-05 | 21 | 34 | ***HLA-DQA2****,* ***HLA-DRB1****,* ***HLA-DQA1****,* ***HLA-DQB1****, HLA-B, HLA-DRB5, HLA-DRA, HLA-DMB, HLA-C, HLA-DMA* |
| KEGG_hsa04640 | Hematopoietic cell lineage | 2.30E-05 | 22 | 79 | ***HLA-DQA2****,* ***HLA-DRB1****,* ***HLA-DQA1****,* ***HLA-DQB1****, MME, TFRC, CD44, TNF, HLA-DRB5, KIT, THPO, HLA-DRA* |
| KEGG_hsa05321 | Inflammatory bowel disease (IBD) | 2.50E-05 | 24 | 54 | ***HLA-DQA2****,* ***HLA-DRB1****,* ***HLA-DQA1****,* ***HLA-DQB1****, TNF, HLA-DRB5, GATA3, HLA-DRA, RORC, MAF, HLA-DMB, IL18R1, IL21R, IL12RB1, HLA-DMA, TGFB1, TLR2* |
| KEGG_hsa04672 | Intestinal immune network for IgA production | 2.60E-05 | 25 | 41 | ***HLA-DQA2****,* ***HLA-DRB1****,* ***HLA-DQA1****,* ***HLA-DQB1****, TNFRSF13C, HLA-DRB5, HLA-DRA, TNFSF13, IL15, ITGB7, HLA-DMB, LTBR, HLA-DMA, TGFB1, CCL25, PIGR* |
| REACTOME_R_HSA_877300 | Interferon gamma signaling | 3.30E-05 | 32 | 85 | ***HLA-DQA2****,* ***HLA-DRB1****,* ***HLA-DQA1****, HLA-DQB2,* ***HLA-DQB1****, HLA-B, OASL, CD44, HLA-DRB5, TRIM10, CAMK2D, HLA-DRA, NCAM1, GBP1, TRIM46, HLA-C, GBP7* |
| KEGG_hsa04940 | Type I diabetes mellitus | 4.20E-05 | 41 | 36 | ***HLA-DQA2****,* ***HLA-DRB1****,* ***HLA-DQA1****,* ***HLA-DQB1****, HLA-B, TNF, HLA-DRB5, HLA-DRA, HLA-DMB, HLA-C, HLA-DMA, PTPRN, LTA, PRF1* |
| KEGG_hsa05323 | Rheumatoid arthritis | 5.30E-05 | 52 | 79 | ***HLA-DQA2****,* ***HLA-DRB1****,* ***HLA-DQA1****,* ***HLA-DQB1****, ATP6V0D2, TNF, HLA-DRB5, ATP6V1B1, CCL2, HLA-DRA, TNFSF13, ATP6V0E2, IL15, HLA-DMB, ATP6V1H, HLA-DMA, CXCL6, TGFB1, TLR2* |
| KEGG_hsa05150 | Staphylococcus aureus infection | 6.40E-05 | 63 | 52 | ***HLA-DQA2****,* ***HLA-DRB1****,* ***HLA-DQA1****,* ***HLA-DQB1****, C2, HLA-DRB5, HLA-DRA, MASP1, HLA-DMB, DSG1, C4B, C4A, HLA-DMA, CFH, FCGR2B* |
| KEGG_hsa04145 | Phagosome | 7.40E-05 | 73 | 139 | ***HLA-DQA2****,* ***HLA-DRB1****,* ***HLA-DQA1****,* ***HLA-DQB1****, HLA-B, TFRC, ATP6V0D2, PLA2R1, CORO1A, TUBA3D, HLA-DRB5, ATP6V1B1, MRC2, HLA-DRA, ATP6V0E2, THBS3, HLA-DMB, TUBB8, HLA-C, ATP6V1H, HLA-DMA, TUBB4A, MARCO, TLR2, TUBA8, EEA1, FCGR2B, DYNC1H1, C1R, CLEC7A, NCF1, THBS2, TUBB6, ITGB1* |
| REACTOME_R_HSA_202424 | Downstream TCR signaling | 8.71E-04 | 870 | 87 | ***HLA-DQA2****,* ***HLA-DRB1****,* ***HLA-DQA1****, HLA-DQB2,* ***HLA-DQB1****, PIK3R2, HLA-DRB5, HLA-DRA, PSMB4, PSMD14, PSMB9, PSMC2, SKP1, PSMB2, PSMF1, PSMD9, NFKB1, PSMB1, PSMB8* |
| KEGG_hsa05164 | Influenza A | 9.83E-04 | 982 | 142 | ***HLA-DQA2****,* ***HLA-DRB1****,* ***HLA-DQA1****,* ***HLA-DQB1****, PRKCB, PIK3R2, HSPA1L, PIK3R3, TNF, HLA-DRB5, HSPA1B, CCL2, PABPN1L, HLA-DRA, RAE1, KPNA1, NLRX1, TMPRSS4, EP300, EIF2AK2, HLA-DMB, HLA-DMA, TLR3, NXT1, IFNAR1, MYD88, NFKB1, RNASEL, MAP2K7, DDX39B, PRSS3, FURIN, FASLG, PRSS1, TYK2, TBK1, IRF7, DDX58, JAK2, MAPK3* |
| KEGG_hsa05140 | Leishmaniasis | 1.23E-03 | 1232 | 65 | ***HLA-DQA2****,* ***HLA-DRB1****,* ***HLA-DQA1****,* ***HLA-DQB1****,* ***PRKCB****, TNF, HLA-DRB5, HLA-DRA, NOS2, HLA-DMB, HLA-DMA, TGFB1, TLR2, MYD88, NFKB1, NCF1, PTPN6, ITGB1* |
| REACTOME_R_HSA_2132295 | MHC class II antigen presentation | 1.45E-03 | 1446 | 93 | ***HLA-DQA2****,* ***HLA-DRB1****,* ***HLA-DQA1****, HLA-DQB2,* ***HLA-DQB1****, AP1S1, HLA-DRB5, AP2S1, KLC4, HLA-DRA, DNM1, KIF2A, HLA-DMB, HLA-DMA, CTSF, DCTN3, DYNC1H1, KIF3C, CTSK, KIF15, DYNLL1, DCTN4, CLTA, KLC1, CTSS, AP1M2* |
| REACTOME_R_HSA_162592 | Integration of provirus | 1.67E-03 | 1601 | 9 | *LIG4, XRCC6, XRCC5, KPNA1, HMGA1* |
| REACTOME_R_HSA_1368082 | RORA activates gene expression | 1.89E-03 | 1887 | 24 | *NRIP1, CHD9, EP300, NR1D1, SMARCD3, NPAS2, MED1, TGS1, TBL1XR1* |
| REACTOME_R_HSA_171052 | LDL-mediated lipid transport | 2.03E-03 | 2023 | 21 | *SOAT1, LDLRAP1, AP2S1, APOB, CETP* |
| KEGG_hsa05168 | Herpes simplex infection | 2.17E-03 | 2174 | 145 | ***HLA-DQA2****,* ***HLA-DRB1****,* ***HLA-DQA1****,* ***HLA-DQB1****, HLA-B, TNF, HLA-DRB5, CCL2, TAF6L, HLA-DRA, EP300, EIF2AK2, IL15, HLA-DMB, HLA-C, UBE2R2, HLA-DMA, SKP1, TLR3, HNRNPK, TNFSF14, TLR2, IFNAR1, TP53, MYD88, LTA, NFKB1, RNASEL, TAF13, FASLG, CSNK2A3, PPP1CC, TBPL1, GTF2IRD1, TYK2, TBK1, IRF7, DDX58, TAF3, JAK2, C3, FADD, IFIT1B* |
| REACTOME_R_HSA_5223345 | Miscellaneous transport and binding events | 2.54E-03 | 2530 | 17 | *PQLC2, ANKH, ADD3, NIPA2, TUSC3* |
| REACTOME_R_HSA_202403 | TCR signaling | 2.98E-03 | 2984 | 106 | ***HLA-DQA2****,* ***HLA-DRB1****,* ***HLA-DQA1****, HLA-DQB2,* ***HLA-DQB1****, PIK3R2, HLA-DRB5, HLA-DRA, PSMB4, PSMD14, PSMB9, PSMC2, SKP1, PSMB2, PSMF1, PSMD9, NFKB1, PSMB1, PAG1, PSMB8* |
| KEGG_hsa04514 | Cell adhesion molecules (CAMs) | 3.05E-03 | 3050 | 124 | ***HLA-DQA2****,* ***HLA-DRB1****,* ***HLA-DQA1****,* ***HLA-DQB1****, HLA-B, ICAM3, HLA-DRB5, SELL, HLA-DRA, NCAM1, CLDN14, ITGB7, HLA-DMB, HLA-C, HLA-DMA, PDCD1LG2, CDH4, NRXN2, OCLN, CLDN18, CD2, CD58, CLDN19, LRRC4C, SIGLEC1, ITGB1, NTNG1, CLDN24, VTCN1, CDH15, CADM3, SELP, CDH5, HLA-E, MADCAM1, GLG1, ICOSLG, HLA-DPB1, CD80* |
| KEGG_hsa00603 | Glycosphingolipid biosynthesis - globo and isoglobo series | 4.24E-03 | 4174 | 13 | *A4GALT, NAGA, B3GALT5, FUT2, FUT1* |
| KEGG_hsa00340 | Histidine metabolism | 5.20E-03 | 5186 | 21 | *FTCD, AMDHD1, ALDH3A1, ALDH1B1, CARNS1, UROC1, ALDH1A3, HAL* |
| REACTOME_R_HSA_913531 | Interferon Signaling | 5.52E-03 | 5519 | 171 | ***HLA-DQA2****,* ***HLA-DRB1****,* ***HLA-DQA1****, HLA-DQB2,* ***HLA-DQB1****, HLA-B, NDC1, OASL, CD44, HLA-DRB5, TRIM10, CAMK2D, HLA-DRA, RAE1, KPNA1, USP18, EIF2AK2, NCAM1, IP6K2, GBP1, TRIM46, HLA-C, NUP214, GBP7, IFNAR1, KPNA4, KPNA5, EIF4A2, RNASEL, NUP155, PSMB8, KPNA7, PTPN6, TRIM45, TYK2, IRF7, TRIM31, DDX58, NUP35, JAK2, MAPK3, IFIT3, IRF6, HLA-E, PML, IRF1, SOCS1, GBP4, TRIM21, CAMK2G, HLA-DPB1* |
| KEGG_hsa04070 | Phosphatidylinositol signaling system | 6.20E-03 | 6201 | 91 | ***PRKCB****, PIK3R2, PIK3R3, CALM1, PLCD1, MTMR6, IMPA1, INPP4A, IP6K2, PIK3C2G, ITPK1, SYNJ2, CALML3, CALML5, DGKH, PLCE1, INPP1, INPPL1* |
| KEGG_hsa05166 | HTLV-I infection | 6.54E-03 | 6540 | 227 | ***HLA-DQA2****,* ***HLA-DRB1****,* ***HLA-DQA1****,* ***HLA-DQB1****, HLA-B, PIK3R2, SMAD4, PIK3R3, TNFRSF13C, TP53INP1, ATF1, TNF, HLA-DRB5, HLA-DRA, BCL2L1, WNT2B, EP300, RANBP1, CTNNB1, POLE3, RRAS, IL15, HLA-DMB, HLA-C, KAT2A, LTBR, HLA-DMA, TGFB1, ANAPC11, FZD3, TP53, CDC27, SLC25A4, CDC23, LTA, WNT2, WNT9B, ANAPC2, POLB, NFKB1, KRAS, ADCY7, RAN, ADCY6, TBPL1, WNT7A, JAK3, DVL1, ANAPC10, NFYB, NFATC4, CDKN2B, CDKN2A, TSPO, TERT, BUB3, HLA-E, RRAS2, TRRAP, CCND2, VDAC2, XBP1, PPP3CA, POLD1, IL2RB, FZD10, HLA-DPB1, SLC2A1* |
| KEGG_hsa_M00089 | Triacylglycerol biosynthesis | 6.58E-03 | 6421 | 11 | *MBOAT2, DGAT2, AGPAT1* |
| REACTOME_R_HSA_164843 | 2-LTR circle formation | 6.73E-03 | 6267 | 7 | *LIG4, XRCC6, XRCC5, HMGA1, XRCC4* |

| **S2 Table. GSEA results from TWAS**  GSEA were performed with total number of imputed protein coding genes from multiple tissues pre-ranked by lung function association p-value.  **more extreme (*n*)** = number of times a better enrichment score was achieved over 1 million random permutations  **size** = number of protein coding genes in the pathway also present in our imputed expression dataset (n=13, 685)  **Genes on the leading edge** = genes by rank responsible for increasing enrichment score (***bold***: consensus genes near GWAS loci; *underlined*: consensus genes outside GWAS loci) | | | | | |
| --- | --- | --- | --- | --- | --- |
| **Pathway ID** | **Description** | **p-value** | **more extreme (*n*)** | **size** | **Genes on the leading edge** |
| **Go Biological Processes** (http://geneontology.org/) | | | | | |
| GO:0050778 | positive regulation of immune response | 5.80E-05 | 57 | 489 | ***HLA-DRB1****, HLA-DRA,* ***HLA-DQA1****,* ***MUC20****,* ***HLA-DQA2****,* ***MUC4****,* ***HLA-DQB1****, NFAM1, CFH,* ***PRKCB****, HLA-DQB2, PIK3AP1, C2, PLEKHA1, BTNL2, ICAM3, HLA-B, TGFB1, DDX60, TNIP2, HMGB1, BAG6, PSMD4, STAT6, YES1, PGLYRP3, CRTAM, MED1, NFKBID, XCL1, MASP1, BCL2, RAET1G, S100A1, HLA-C, TNFRSF13C, PSMD6, PRKCQ, CLEC7A, CFB, HLA-DRB5, PRKCZ, CD38, FGG, C4B, KRAS, PELI1, LIME1, TLR2, PRKCH, MYO10, PIK3C3, TLR3, IL33, PSMA7, ARPC4, APPL2, IL13, CTSK, PAK2, PSME4, RAET1E, BTN3A3, FGR, SKAP1, FYN, DDX1, CTSS, MUC16, PLA2G1B, VTCN1, SPG21, LRRC19, PSMB1, HEXIM1, TRAF6, TNFSF13, IDO1, ULBP3, ULBP1, C4A, PSMD7, CFHR1, IKBKE, RPS19, THEMIS2, C5AR1, GBP5, CD4, SH2B2, EP300, FLOT1, XRCC6, GRB2, LBP, FZD5, ULBP2, FGB, DHX36, NFKBIZ, CD80, DDX58, MLH1, IRAK3, XRCC5, IL15, PSMF1, IL27RA, TNFSF4, RIOK3, PSMD11, PYHIN1, CD79A, S100A14, HK1, VAMP8, IKBKB, P2RX7, PGC, RGCC, AIM2, PSMD3, DENND1B, THY1, PSMA6, ARPC1A, PSMD9, IL12A, ELMO2, TGFB2, PIK3R2, ZC3H12A, DHX58, WNK1, HLX, MAVS, TRAF3, VAV3, MALT1, BTNL8, PIK3CA, MARCO, MAPK3, ACTR2, ICOSLG, CD36, PRAM1, TNFRSF21, SLC11A1, SKP1, CD14, MASP2, SLA2, HLA-DMB, PRKD2, TANK, CRKL, ABL1, CCL5, PSMB9, BTNL3, RC3H2, LILRA2, ADAM8, MUC3A, ATAD5, C9, TIRAP, DOCK1, MUC1* |
| GO:0060333 | interferon-gamma-mediated signaling pathway | 6.20E-05 | 61 | 52 | ***HLA-DRB1****, HLA-DRA,* ***HLA-DQA1****,* ***HLA-DQA2****,* ***HLA-DQB1****, OASL, HLA-DQB2, CD44, HLA-B, HLA-C, HLA-DRB5* |
| GO:0018230 | peptidyl-L-cysteine S-palmitoylation | 6.63E-05 | 65 | 18 | ***ZDHHC11****,* ***ZDHHC11B****, GOLGA7* |
| GO:0002253 | activation of immune response | 7.90E-05 | 78 | 337 | ***HLA-DRB1****, HLA-DRA,* ***HLA-DQA1****,* ***MUC20****,* ***HLA-DQA2****,* ***MUC4****,* ***HLA-DQB1****, CFH,* ***PRKCB****, HLA-DQB2, PIK3AP1, C2, PLEKHA1, BTNL2, ICAM3, TNIP2, HMGB1, BAG6, PSMD4, YES1, PGLYRP3, NFKBID, MASP1, BCL2, S100A1, PSMD6, PRKCQ, CLEC7A, CFB, HLA-DRB5, CD38, FGG, C4B, KRAS, LIME1, TLR2, MYO10, PIK3C3, TLR3, PSMA7, ARPC4, CTSK, PAK2, PSME4, BTN3A3, FGR, SKAP1, FYN, CTSS, MUC16, VTCN1, SPG21, LRRC19, PSMB1, HEXIM1, TRAF6, C4A, PSMD7, CFHR1, IKBKE, THEMIS2, C5AR1, CD4, SH2B2, EP300, XRCC6, GRB2, LBP, FGB, NFKBIZ, DDX58, IRAK3, XRCC5, PSMF1, PSMD11, PYHIN1, CD79A, S100A14, IKBKB, RGCC, AIM2, PSMD3, DENND1B, THY1, PSMA6, ARPC1A, PSMD9, ELMO2, PIK3R2, ZC3H12A, WNK1, MAVS, TRAF3, VAV3, MALT1, BTNL8, PIK3CA, MARCO, MAPK3, ACTR2, ICOSLG, CD36, PRAM1, TNFRSF21, SKP1, CD14, MASP2, SLA2, PRKD2, TANK, CRKL, ABL1, PSMB9, BTNL3, RC3H2, LILRA2, MUC3A, C9, TIRAP, DOCK1, MUC1* |
| GO:0050851 | antigen receptor-mediated signaling pathway | 9.70E-05 | 96 | 133 | ***HLA-DRB1****, HLA-DRA,* ***HLA-DQA1****,* ***HLA-DQA2****,* ***HLA-DQB1****,* ***PRKCB****, HLA-DQB2, PLEKHA1, BTNL2, PSMD4, NFKBID, BCL2, PSMD6, PRKCQ, HLA-DRB5, CD38, LIME1, PSMA7, PAK2, PSME4, BTN3A3, SKAP1, FYN, VTCN1, SPG21, PSMB1, TRAF6, PSMD7, THEMIS2, CD4, SH2B2, NFKBIZ, PSMF1, PSMD11, CD79A, IKBKB, PSMD3, DENND1B, THY1, PSMA6, PSMD9, PIK3R2, ZC3H12A, WNK1, VAV3, MALT1, BTNL8, PIK3CA, ICOSLG, PRAM1, TNFRSF21, SKP1, SLA2, PRKD2, CRKL, ABL1, PSMB9, BTNL3, RC3H2* |
| GO:0002757 | immune response-activating signal transduction | 3.83E-04 | 382 | 297 | ***HLA-DRB1****, HLA-DRA,* ***HLA-DQA1****,* ***MUC20****,* ***HLA-DQA2****,* ***MUC4****,* ***HLA-DQB1****,* ***PRKCB****, HLA-DQB2, PIK3AP1, PLEKHA1, BTNL2, ICAM3, TNIP2, HMGB1, BAG6, PSMD4, YES1, PGLYRP3, NFKBID, BCL2, S100A1, PSMD6, PRKCQ, CLEC7A, HLA-DRB5, CD38, FGG, KRAS, LIME1, TLR2, MYO10, PIK3C3, TLR3, PSMA7, ARPC4, CTSK, PAK2, PSME4, BTN3A3, FGR, SKAP1, FYN, CTSS, MUC16, VTCN1, SPG21, LRRC19, PSMB1, TRAF6, PSMD7, IKBKE, THEMIS2, C5AR1, CD4, SH2B2, EP300, GRB2, LBP, FGB, NFKBIZ, DDX58, IRAK3, PSMF1, PSMD11, CD79A, S100A14, IKBKB, PSMD3, DENND1B, THY1, PSMA6, ARPC1A, PSMD9, ELMO2, PIK3R2, ZC3H12A, WNK1, TRAF3, VAV3, MALT1, BTNL8, PIK3CA, MARCO, MAPK3, ACTR2, ICOSLG, CD36, PRAM1, TNFRSF21, SKP1, CD14, SLA2, PRKD2, TANK, CRKL, ABL1, PSMB9, BTNL3, RC3H2, LILRA2, MUC3A, TIRAP, DOCK1, MUC1* |
| GO:0002764 | immune response-regulating signaling pathway | 4.31E-04 | 430 | 322 | ***HLA-DRB1****, HLA-DRA,* ***HLA-DQA1****,* ***MUC20****,* ***HLA-DQA2****,* ***MUC4****,* ***HLA-DQB1****,* ***PRKCB****, HLA-DQB2, PIK3AP1, PLEKHA1, BTNL2, ICAM3, TNIP2, HMGB1, BAG6, PSMD4, YES1, PGLYRP3, NFKBID, BCL2, S100A1, PSMD6, PRKCQ, CLEC7A, HLA-DRB5, LILRA4, CD38, FGG, KRAS, LIME1, TLR2, MYO10, PIK3C3, TLR3, PSMA7, ARPC4, CALM1, CTSK, PAK2, PSME4, BTN3A3, FGR, SKAP1, FYN, CTSS, MUC16, VTCN1, SPG21, LRRC19, PSMB1, TRAF6, PSMD7, MAPK9, IKBKE, THEMIS2, C5AR1, CD4, SH2B2, EP300, GRB2, LBP, FGB, NFKBIZ, DDX58, IRAK3, PSMF1, PSMD11, CD79A, S100A14, PPP3CA, IKBKB, PSMD3, DENND1B, THY1, PSMA6, FER, ARPC1A, PSMD9, ELMO2, PIK3R2, ZC3H12A, WNK1, TRAF3, VAV3, MALT1, BTNL8, PIK3CA, MARCO, MAPK3, ACTR2, ICOSLG, CD36, PRAM1, TNFRSF21, SKP1, CD14, SLA2, PRKD2, TANK, CRKL, SOS1, ABL1, PSMB9, BTNL3, RC3H2, LILRA2, MUC3A, TIRAP, DOCK1, MUC1* |
| GO:1904779 | regulation of protein localization to centrosome | 4.68E-04 | 453 | 10 | ***CEP72****, MARK4* |
| GO:0002768 | immune response-regulating cell surface receptor signaling pathway | 4.81E-04 | 480 | 238 | ***HLA-DRB1****, HLA-DRA,* ***HLA-DQA1****,* ***MUC20****,* ***HLA-DQA2****,* ***MUC4****,* ***HLA-DQB1****,* ***PRKCB****, HLA-DQB2, PLEKHA1, BTNL2, ICAM3, BAG6, PSMD4, YES1, NFKBID, BCL2, PSMD6, PRKCQ, HLA-DRB5, LILRA4, CD38, KRAS, LIME1, MYO10, PSMA7, ARPC4, CALM1, PAK2, PSME4, BTN3A3, FGR, SKAP1, FYN, MUC16, VTCN1, SPG21, PSMB1, TRAF6, PSMD7, MAPK9, THEMIS2, C5AR1, CD4, SH2B2, EP300, GRB2, NFKBIZ, PSMF1, PSMD11, CD79A, PPP3CA, IKBKB, PSMD3, DENND1B, THY1, PSMA6, FER, ARPC1A, PSMD9, ELMO2, PIK3R2, ZC3H12A, WNK1, VAV3, MALT1, BTNL8, PIK3CA, MAPK3, ACTR2, ICOSLG, PRAM1, TNFRSF21, SKP1, SLA2, PRKD2, CRKL, SOS1, ABL1, PSMB9, BTNL3, RC3H2, LILRA2, MUC3A, DOCK1, MUC1* |
| GO:0002429 | immune response-activating cell surface receptor signaling pathway | 4.86E-04 | 485 | 215 | ***HLA-DRB1****, HLA-DRA,* ***HLA-DQA1****,* ***MUC20****,* ***HLA-DQA2****,* ***MUC4****,* ***HLA-DQB1****,* ***PRKCB****, HLA-DQB2, PLEKHA1, BTNL2, ICAM3, BAG6, PSMD4, YES1, NFKBID, BCL2, PSMD6, PRKCQ, HLA-DRB5, CD38, KRAS, LIME1, MYO10, PSMA7, ARPC4, PAK2, PSME4, BTN3A3, FGR, SKAP1, FYN, MUC16, VTCN1, SPG21, PSMB1, TRAF6, PSMD7, THEMIS2, C5AR1, CD4, SH2B2, EP300, GRB2, NFKBIZ, PSMF1, PSMD11, CD79A, IKBKB, PSMD3, DENND1B, THY1, PSMA6, ARPC1A, PSMD9, ELMO2, PIK3R2, ZC3H12A, WNK1, VAV3, MALT1, BTNL8, PIK3CA, MAPK3, ACTR2, ICOSLG, PRAM1, TNFRSF21, SKP1, SLA2, PRKD2, CRKL, ABL1, PSMB9, BTNL3, RC3H2, LILRA2, MUC3A, DOCK1, MUC1* |
| GO:0018345 | protein palmitoylation | 5.04E-04 | 503 | 27 | ***ZDHHC11****,* ***ZDHHC11B****, ZDHHC5, ZDHHC16, DBI* |
| GO:0050852 | T cell receptor signaling pathway | 5.66E-04 | 565 | 117 | ***HLA-DRB1****, HLA-DRA,* ***HLA-DQA1****,* ***HLA-DQA2****,* ***HLA-DQB1****, HLA-DQB2, BTNL2, PSMD4, NFKBID, PSMD6, PRKCQ, HLA-DRB5, LIME1, PSMA7, PAK2, PSME4, BTN3A3, SKAP1, FYN, VTCN1, PSMB1, TRAF6, PSMD7, THEMIS2, CD4, NFKBIZ, PSMF1, PSMD11, IKBKB, PSMD3, DENND1B, THY1, PSMA6, PSMD9, PIK3R2, ZC3H12A, WNK1, MALT1, BTNL8, PIK3CA, ICOSLG, PRAM1, TNFRSF21, SKP1, PRKD2, CRKL, ABL1, PSMB9, BTNL3, RC3H2* |
| GO:2000027 | regulation of animal organ morphogenesis | 7.91E-04 | 790 | 175 | *FOXP2, FGFR2, TGFB1, PTN, PSMD4, TBX5, MED1, EDN1, BCL2, PSMD6, FZD3, SFRP2, NOTCH1, SIX4, STOX1, FZD1, SIX1, RUNX2, DKK1, DMRT3, HGF, TWIST1, PSMA7, PSME4, WNT9B, SMAD4, WNT11, NTN4, BMPR2, TGFBR1, PSMB1, LGR4, WNT7A, SAPCD2, SP6, CARM1, PSMD7, CSF1, GLI1, SMURF1, ASPN, RNF207, VEGFA, EYA1, GNG5, SOX9, FZD5, PSMF1, FZD7, ROR1, PSMD11, SFRP1, STAT1, RBPJ, WNT2B, TBX1, POR, PSMD3, SOX8, AP2S1, PSMA6, FGF7, VDR, PSMD9, TGFB2, PRICKLE2, ESR1, FGF1, BMPR1A, HOXB7* |
| GO:0048002 | antigen processing and presentation of peptide antigen | 7.93E-04 | 792 | 132 | ***HLA-DRB1****, HLA-DRA,* ***HLA-DQA1****,* ***HLA-DQA2****,* ***HLA-DQB1****, HLA-DQB2, CAPZA2, HLA-B, KIF3B, PSMD4, AP1M2, HLA-C, PSMD6, HLA-DRB5, PSMA7, PSME4, SEC23A, CTSS, NCF1, PSMB1, TRAF6, KIF2A, PSMD7, SPTBN2, CD207, RACGAP1, CTSF, TAP1, PSMF1, PSMD11, AP1M1, VAMP8, IKBKB, KLC2, PSMD3, AP2S1, PSMA6, PSMD9, VAMP3, CAPZA3, SEC24C, AP2B1, CD36, SLC11A1, PDIA3, HLA-DMB, PSMB9* |
| GO:0019221 | cytokine-mediated signaling pathway | 1.51E-03 | 1508 | 427 | ***HLA-DRB1****, HLA-DRA,* ***HLA-DQA1****,* ***HLA-DQA2****,* ***HLA-DQB1****, LCN2, RORC, OASL, HLA-DQB2, CD44, CXCL2, PF4, HLA-B, TGFB1, TNIP2, COMMD7, PSMD4, GREM2, NOS2, CXCL6, STAT6, CCL4L1, OXSR1, ZC3H15, CDIP1, CCL26, XCL1, TNFRSF8, BCL2, HLA-C, TNFRSF13C, PSMD6, SOCS1, PTPRZ1, HLA-DRB5, GPR75, TSLP, BCL2L1, KRAS, PELI1, HGF, LTBR, MTAP, TWIST1, IL33, PSMA7, APPL2, IRS1, IL13, CCL22, PAK2, PSME4, PF4V1, SMAD4, FYN, IL37, IFNAR1, CSNK2B, RBM15, ADIPOR1, ACKR2, CSF3, PSMB1, TALDO1, LMNB1, PLCB1, CAMK2G, HIF1A, TRAF6, TNFSF13, TNFRSF6B, IL1RL2, BRWD1, TNFRSF17, RPS6KA4, PSMD7, P4HB, OAS3, CSF1, LRP8, OPRM1, NCAM1, CD4, VEGFA, SH2B2, IL22RA1, TNFSF9, IL1RAP, BAD, GRB2, LBP, CCL3L1, CD80, RPLP0, IRAK3, IL15, ZEB1, PSMF1, IL27RA, TNFSF4, CXCL12, PSMD11, ALOX15, STAT1, KRT8, S1PR1, TCP1, TOLLIP, IKBKB, PML, AIM2, PDCD4, XCL2, PSMD3, CCL13, PSMA6, FER, EDN2, PSMD9, IL12A, RALA, BOLA2, JAGN1, SMARCA4, SNRPA1, WNK1, ADIPOR2, TNFSF12, TRAF3, IRF6, EPOR, IFNGR2, PIK3CA, SOD1, CBL, MAPK3, CXCL13, ICAM1, MMP2, CD36, IFNLR1, CNTFR, SKP1, CRKL, SOS1, CCL5, MMP1, PSMB9, IRF1, EPO, RNASEL, CSF2RB, MUC1* |
| GO:0007099 | centriole replication | 1.66E-03 | 1620 | 11 | ***CEP72****, CENPJ* |
| GO:0019882 | antigen processing and presentation | 1.93E-03 | 1929 | 154 | ***HLA-DRB1****, HLA-DRA,* ***HLA-DQA1****,* ***HLA-DQA2****,* ***HLA-DQB1****, HLA-DQB2, CAPZA2, HLA-B, KIF3B, PSMD4, AP1M2, HLA-C, PSMD6, HLA-DRB5, PSMA7, RAB4A, PSME4, SEC23A, CTSS, NCF1, PSMB1, TRAF6, AP3D1, AP3B1, KIF2A, PSMD7, SPTBN2, CD207, RACGAP1, CTSF, TAP1, PSMF1, PSMD11, AP1M1, VAMP8, IKBKB, KLC2, PSMD3, AP2S1, PSMA6, PSMD9, VAMP3, CAPZA3, SEC24C, ICAM1, AP2B1, CD36, SLC11A1, PDIA3, HLA-DMB, PSMB9* |
| GO:0048012 | hepatocyte growth factor receptor signaling pathway | 2.02E-03 | 1957 | 10 | *MET,* ***MUC20,*** *STMN1, HGF* |
| GO:0006105 | succinate metabolic process | 2.09E-03 | 1900 | 6 | *SDHA, SUCLA2* |
| GO:0006605 | protein targeting | 2.19E-03 | 2194 | 232 | ***ZDHHC11****,* ***ZDHHC11B****, TIMM10, RPL7A, EXOC4, RPL18, AP1M2, NOS2, CAT, SCARB2, NCOA4, PMM1, RASSF9, ATG4C, FBXO7, MFF, ZFAND6, SLC27A2, MFN2, PEX12, PARD3, NCF1, ACOX2, RPL10A, PRKCI, EPHX2, HOMER3, RPS24, SEC63, AMACR, AP3D1, NUP54, AP3B1, SNX16, PEX26, TAOK2, RPS7, RPS19, SMURF1, RPL31, PECR, GOLGA7, RPLP0, ACOT2, GOSR2, DDO, RPL4, TSPO, VPS13D, RAB3IP, SORL1, NUDT7, RPL27A* |
| GO:0002377 | immunoglobulin production | 2.60E-03 | 2365 | 6 | ***HLA-DQB1****, IL7R* |
| GO:0007143 | female meiotic nuclear division | 2.92E-03 | 2835 | 10 | *TRIP13, CDC25B* |
| GO:0090238 | positive regulation of arachidonic acid secretion | 3.25E-03 | 2856 | 5 | *PLA2R1, MIF* |
| GO:1904424 | regulation of GTP binding | 3.35E-03 | 3251 | 10 | *MET, STMN1* |
| GO:0031643 | positive regulation of myelination | 3.80E-03 | 3749 | 13 | *MYRF, TENM4, HGF, S100B, PARD3* |
| GO:0007179 | transforming growth factor beta receptor signaling pathway | 4.17E-03 | 4174 | 74 | *ADAM9, ZYX, SMAD1, TGFB1, AMHR2, NRROS, GDF9, PRKCZ, NODAL, SKI, CDH5, SMAD3, APPL2, SMAD4, BMPR2, TGFBR1, PARD3, PTPRK, ACVRL1, LTBP4, TGFBRAP1, SMURF1, FNTA, MSTN, HIPK2, FURIN, DUSP15, ACVR1B, PML, TGFB2, BMPR1A, CBL, GDF15, ARRB2, GDF5* |
| GO:0048753 | pigment granule organization | 4.39E-03 | 4379 | 20 | *BCL2, TRAPPC6A, RAB38, BLOC1S3, SNAPIN, AP3D1, AP3B1, ASIP, AP1M1* |
| GO:0098534 | centriole assembly | 4.56E-03 | 4525 | 15 | ***CEP72****, MCIDAS* |
| GO:0007178 | transmembrane receptor protein serine/threonine kinase signaling pathway | 4.79E-03 | 4792 | 117 | *ADAM9, ZYX, SMAD1, TGFB1, AMHR2, GREM2, NRROS, GDF9, PRKCZ, NODAL, RUNX2, SKI, CDH5, BMP6, SMAD3, APPL2, SMAD4, BMPR2, TGFBR1, PARD3, PTPRK, ACVRL1, LTBP4, TGFBRAP1, BMP8A, SMURF1, FNTA, MSTN, HIPK2, FURIN, RGMA, DUSP15, RYR2, ACVR1B, PML, SMPD3, PDCD4, COMP, CER1, TGFB2, RGMB, BMPR1A, BMP8B, DLX5, CBL, MAPK3, LEF1, GDF15, ARRB2, GDF5* |
| GO:0070193 | synaptonemal complex organization | 4.87E-03 | 4813 | 14 | *TRIP13, BAG6, SYCE1, UBE2B, AGO4, HORMAD1, PLK1* |
| GO:0007007 | inner mitochondrial membrane organization | 4.88E-03 | 4764 | 11 | *TIMM10, SAMM50* |
| GO:0031047 | gene silencing by RNA | 4.90E-03 | 4895 | 34 | *PIWIL1, NCBP2, CNOT1, HELZ, TDRKH, MOV10L1, NRDE2, CNOT2, AGO4, EIF6, PIWIL4, TNRC6A, PABPC1, FKBP6* |
| GO:0002476 | antigen processing and presentation of endogenous peptide antigen via MHC class Ib | 5.19E-03 | 4724 | 6 | *HLA-B, HLA-C* |
| GO:0007130 | synaptonemal complex assembly | 6.51E-03 | 6357 | 11 | *TRIP13, BAG6, SYCE1, AGO4, HORMAD1* |
| GO:0034629 | cellular protein-containing complex localization | 6.88E-03 | 6867 | 22 | ***CEP72****,* ***EXOC3****, DLG1, SNAPIN, EXOC8, MIOS* |
| GO:0033313 | meiotic cell cycle checkpoint | 7.19E-03 | 6543 | 6 | *TRIP13, HORMAD1* |
| GO:0071712 | ER-associated misfolded protein catabolic process | 7.38E-03 | 7087 | 9 | *BAG6, RNF5, TOR1A, VCP, DERL1* |
| GO:0030514 | negative regulation of BMP signaling pathway | 7.50E-03 | 7500 | 33 | *TMPRSS6, GREM2, SOST, SFRP2, NOTCH1, FZD1, DKK1, SKI, PPM1A, SMURF1, HIPK2, SFRP1, NBL1, VWC2, CER1, TRIM33* |
| GO:0006612 | protein targeting to membrane | 7.50E-03 | 7504 | 101 | ***ZDHHC11****,* ***ZDHHC11B****, RPL7A, EXOC4, RPL18, ATG4C, PARD3, NCF1, RPL10A, PRKCI, RPS24, SEC63, PEX26, TAOK2, RPS7, RPS19, RPL31, GOLGA7, RPLP0, RPL4, RAB3IP, RPL27A* |
| GO:0002495 | antigen processing and presentation of peptide antigen via MHC class II | 7.61E-03 | 7607 | 74 | ***HLA-DRB1****, HLA-DRA,* ***HLA-DQA1****,* ***HLA-DQA2****,* ***HLA-DQB1****, HLA-DQB2, CAPZA2, KIF3B, AP1M2, HLA-DRB5, SEC23A, CTSS, TRAF6, KIF2A, SPTBN2, RACGAP1, CTSF, AP1M1, KLC2, AP2S1* |
| GO:0019886 | antigen processing and presentation of exogenous peptide antigen via MHC class II | 7.61E-03 | 7607 | 74 | ***HLA-DRB1****, HLA-DRA,* ***HLA-DQA1****,* ***HLA-DQA2****,* ***HLA-DQB1****, HLA-DQB2, CAPZA2, KIF3B, AP1M2, HLA-DRB5, SEC23A, CTSS, TRAF6, KIF2A, SPTBN2, RACGAP1, CTSF, AP1M1, KLC2, AP2S1* |
| GO:0002504 | antigen processing and presentation of peptide or polysaccharide antigen via MHC class II | 7.61E-03 | 7607 | 74 | ***HLA-DRB1****, HLA-DRA,* ***HLA-DQA1****,* ***HLA-DQA2****,* ***HLA-DQB1****, HLA-DQB2, CAPZA2, KIF3B, AP1M2, HLA-DRB5, SEC23A, CTSS, TRAF6, KIF2A, SPTBN2, RACGAP1, CTSF, AP1M1, KLC2, AP2S1* |
| GO:0050861 | positive regulation of B cell receptor signaling pathway | 8.02E-03 | 7606 | 8 | *NFAM1,* ***PRKCB****, PRKCH* |
|  |  |  |  |  |  |
| **Biosystems** (https://www.ncbi.nlm.nih.gov/biosystems/) | | | | | |
| KEGG_hsa05320 | Autoimmune thyroid disease | 7.00E-06 | 6 | 25 | ***HLA-DRB1****, HLA-DRA,* ***HLA-DQA1****,* ***HLA-DQA2****,* ***HLA-DQB1****, HLA-B, HLA-C, HLA-DRB5* |
| KEGG_hsa05332 | Graft-versus-host disease | 1.80E-05 | 17 | 25 | ***HLA-DRB1****, HLA-DRA,* ***HLA-DQA1****,* ***HLA-DQA2****,* ***HLA-DQB1****, HLA-B, HLA-C, HLA-DRB5* |
| KEGG_hsa05330 | Allograft rejection | 1.90E-05 | 18 | 23 | ***HLA-DRB1****, HLA-DRA,* ***HLA-DQA1****,* ***HLA-DQA2****,* ***HLA-DQB1****, HLA-B, HLA-C, HLA-DRB5* |
| KEGG_hsa05150 | Staphylococcus aureus infection | 3.70E-05 | 36 | 37 | ***HLA-DRB1****, HLA-DRA,* ***HLA-DQA1****,* ***HLA-DQA2****,* ***HLA-DQB1****, CFH, C2, MASP1, CFB, HLA-DRB5, FGG, C4B* |
| KEGG_hsa05416 | Viral myocarditis | 6.70E-05 | 66 | 49 | ***HLA-DRB1****, HLA-DRA,* ***HLA-DQA1****,* ***HLA-DQA2****,* ***HLA-DQB1****, RAC2, HLA-B, HLA-C, ABL2, HLA-DRB5, CXADR, FYN* |
| KEGG_hsa04940 | Type I diabetes mellitus | 7.50E-05 | 74 | 29 | ***HLA-DRB1****, HLA-DRA,* ***HLA-DQA1****,* ***HLA-DQA2****,* ***HLA-DQB1****, HLA-B, HLA-C, HLA-DRB5* |
| KEGG_hsa04612 | Antigen processing and presentation | 8.20E-05 | 81 | 47 | ***HLA-DRB1****, HLA-DRA,* ***HLA-DQA1****,* ***HLA-DQA2****,* ***HLA-DQB1****, HLA-B, HSPA1L, HLA-C, HLA-DRB5* |
| REACTOME_R_HSA_202430 | Translocation of ZAP-70 to Immunological synapse | 1.04E-04 | 101 | 12 | ***HLA-DRB1****, HLA-DRA,* ***HLA-DQA1****,* ***HLA-DQA2****,* ***HLA-DQB1****, HLA-DQB2, HLA-DRB5* |
| KEGG_hsa05310 | Asthma | 1.13E-04 | 112 | 21 | ***HLA-DRB1****, HLA-DRA,* ***HLA-DQA1****,* ***HLA-DQA2****,* ***HLA-DQB1****, PRG2, HLA-DRB5, RNASE3, IL13* |
| REACTOME_R_HSA_389948 | PD-1 signaling | 1.41E-04 | 138 | 13 | ***HLA-DRB1****, HLA-DRA,* ***HLA-DQA1****,* ***HLA-DQA2****,* ***HLA-DQB1****, HLA-DQB2, PDCD1LG2, HLA-DRB5* |
| KEGG_hsa05321 | Inflammatory bowel disease | 2.29E-04 | 228 | 43 | ***HLA-DRB1****, HLA-DRA,* ***HLA-DQA1****,* ***HLA-DQA2****,* ***HLA-DQB1****, RORC, TGFB1, STAT6, HLA-DRB5, TLR2, SMAD3, IL13* |
| REACTOME_R_HSA_202427 | Phosphorylation of CD3 and TCR zeta chains | 2.90E-04 | 286 | 14 | ***HLA-DRB1****, HLA-DRA,* ***HLA-DQA1****,* ***HLA-DQA2****,* ***HLA-DQB1****, HLA-DQB2, HLA-DRB5* |
| REACTOME_R_HSA_202433 | Generation of second messenger molecules | 4.10E-04 | 408 | 21 | ***HLA-DRB1****, HLA-DRA,* ***HLA-DQA1****,* ***HLA-DQA2****,* ***HLA-DQB1****, HLA-DQB2, HLA-DRB5, PAK2* |
| KEGG_hsa05323 | Rheumatoid arthritis | 4.80E-04 | 479 | 62 | ***HLA-DRB1****, HLA-DRA,* ***HLA-DQA1****,* ***HLA-DQA2****,* ***HLA-DQB1****, TGFB1, ATP6V1B1, CXCL6, TEK, ATP6V0E2, HLA-DRB5, ATP6V1G2, ATP6V1C2, TLR2, CTSK, ANGPT1, TNFSF13, CSF1, VEGFA, CCL3L1, CD80, IL15, CXCL12, ATP6V1G1* |
| KEGG_hsa04145 | Phagosome | 7.18E-04 | 717 | 112 | ***HLA-DRB1****, HLA-DRA,* ***HLA-DQA1****,* ***HLA-DQA2****,* ***HLA-DQB1****, PLA2R1, EEA1, HLA-B, ATP6V1B1, TUBA3D, HLA-C, CLEC7A, ATP6V0E2, HLA-DRB5, ATP6V1G2, ATP6V1C2, MRC2, TLR2, PIK3C3, SCARB1, CTSS, NCF1, TUBAL3* |
| KEGG_hsa04659 | Th17 cell differentiation | 9.79E-04 | 978 | 67 | ***HLA-DRB1****, HLA-DRA,* ***HLA-DQA1****,* ***HLA-DQA2****,* ***HLA-DQB1****, RORC, TGFB1, STAT6, PRKCQ, HLA-DRB5, SMAD3, SMAD4, TGFBR1, IL17D, HIF1A, MAPK9, CD4, MAPK12, IL1RAP, IL27RA, STAT1, PPP3CA, IKBKB* |
| REACTOME_R_HSA_877300 | Interferon gamma signaling | 1.03E-03 | 1031 | 67 | ***HLA-DRB1****, HLA-DRA,* ***HLA-DQA1****,* ***HLA-DQA2****,* ***HLA-DQB1****, OASL, HLA-DQB2, CD44, HLA-B, HLA-C, SOCS1, HLA-DRB5* |
| Pathway Interaction Database_ecadherin_stabilization_pathway | Stabilization and expansion of the E-cadherin adherens junction | 1.05E-03 | 1053 | 37 | ***EXOC3****, MET, ZYX, EXOC4, LIMA1, CAMSAP3, GIT1, HGF* |
| REACTOME_R_HSA_202424 | Downstream TCR signaling | 1.15E-03 | 1148 | 61 | ***HLA-DRB1****, HLA-DRA,* ***HLA-DQA1****,* ***HLA-DQA2****,* ***HLA-DQB1****, HLA-DQB2, PSMD4, PSMD6, PRKCQ, HLA-DRB5, PSMA7, PSME4, PSMB1, TRAF6, PSMD7, CD4, PSMF1, PSMD11, IKBKB, PSMD3, PSMA6, PSMD9, PIK3R2, CDC34, MALT1, PIK3CA, SKP1* |
| KEGG_hsa04672 | Intestinal immune network for IgA production | 1.77E-03 | 1773 | 30 | ***HLA-DRB1****, HLA-DRA,* ***HLA-DQA1****,* ***HLA-DQA2****,* ***HLA-DQB1****, TGFB1, TNFRSF13C, HLA-DRB5, LTBR, TNFSF13, TNFRSF17, CD80, IL15, CXCL12* |
| KEGG_hsa05140 | Leishmaniasis | 2.01E-03 | 2009 | 49 | ***HLA-DRB1****, HLA-DRA,* ***HLA-DQA1****,* ***HLA-DQA2****,* ***HLA-DQB1****,* ***PRKCB****, TGFB1, NOS2, HLA-DRB5, TLR2* |
| KEGG_hsa05322 | Systemic lupus erythematosus | 2.19E-03 | 2193 | 52 | ***HLA-DRB1****, HLA-DRA,* ***HLA-DQA1****,* ***HLA-DQA2****,* ***HLA-DQB1****, C2, HLA-DRB5, C4B, H2AFJ, H2AFV, C4A, HIST1H2AE, H3F3B, HIST1H2BD, CD80, SNRPB* |
| REACTOME_R_HSA_388841 | Costimulation by the CD28 family | 2.25E-03 | 2251 | 47 | ***HLA-DRB1****, HLA-DRA,* ***HLA-DQA1****,* ***HLA-DQA2****,* ***HLA-DQB1****, HLA-DQB2, PDCD1LG2, YES1, HLA-DRB5, PIK3R3, PAK2, FYN, CD4, GRB2, CD80, PPP2CB* |
| REACTOME_R_HSA_6803157 | Antimicrobial peptides | 2.34E-03 | 2338 | 34 | *BPIFB1, LCN2, DEFB124, PGLYRP3, LEAP2, BPIFB4, RNASE3, TLR2, ART1, CHGA* |
| KEGG_hsa04514 | Cell adhesion molecules | 3.11E-03 | 3112 | 106 | ***HLA-DRB1****, HLA-DRA,* ***HLA-DQA1****,* ***HLA-DQA2****,* ***HLA-DQB1****, ICAM3, HLA-B, PDCD1LG2, HLA-C, CDH4, HLA-DRB5, NRXN2, MPZL1, CDH5, SDC3, SELL, VTCN1, SELP, LRRC4B, PTPRM, NCAM1, CNTNAP2, CD4, CNTN2, CD80, NRXN1* |
| KEGG_hsa05168 | Herpes simplex infection | 3.95E-03 | 3946 | 105 | ***HLA-DRB1****, HLA-DRA,* ***HLA-DQA1****,* ***HLA-DQA2****,* ***HLA-DQB1****, HLA-B, TAF3, HLA-C, MCRS1, HLA-DRB5, EIF2AK1, TAF13, TLR2, TLR3, IFNAR1, CSNK2B, TRAF6, MAPK9, OAS3, IKBKE, EP300, DDX58, CDK1, TAP1, IL15, UBE2R2, STAT1, IKBKB, PML, PPP1CC, TAF5L, IL12A, MAVS, TRAF3, IFNGR2, CDC34, SKP1, HLA-DMB, CCL5* |
| REACTOME_R_HSA_202403 | TCR signaling | 4.05E-03 | 4050 | 76 | ***HLA-DRB1****, HLA-DRA,* ***HLA-DQA1****,* ***HLA-DQA2****,* ***HLA-DQB1****, HLA-DQB2, PSMD4, PSMD6, PRKCQ, HLA-DRB5, PSMA7, PAK2, PSME4, PSMB1, TRAF6, PSMD7, CD4, PSMF1, PSMD11, IKBKB, PSMD3, PSMA6, PSMD9, PIK3R2, CDC34, MALT1, PIK3CA, ENAH, VASP, SKP1* |
| KEGG_hsa05169 | Epstein-Barr virus infection | 5.60E-03 | 5598 | 144 | ***HLA-DRB1****, HLA-DRA,* ***HLA-DQA1****,* ***HLA-DQA2****,* ***HLA-DQB1****, CD44, HLA-B, PSMD4, POLR2K, HSPA1L, BCL2, HLA-C, PSMD6, HLA-DRB5, CD38, EIF2AK1, ENTPD1, PIK3R3, FGR, CSNK2B, TRAF6, NUP214, CCNA2, PSMD7, MAPK9, MAPK12, ADRM1, EP300, GTF2E2, DDX58, CDK1, PSMD11, RBPJ, POLR2I, CD58, IKBKB, HDAC5, PSMD3, POLR2H, PIK3R2, TRAF3, PIK3CA, PTMA, ICAM1* |
| REACTOME_R_HSA_400685 | Sema4D in semaphorin signaling | 5.98E-03 | 5969 | 22 | *MET, RAC2, MYH9, ERBB2, RHOC, MYH14, ARHGEF11* |
| REACTOME_R_HSA_983169 | Class I MHC mediated antigen processing & presentation | 6.27E-03 | 6267 | 269 | *GAN, MEX3C, FBXL7, MKRN1, HLA-B, DTX3L, BLMH, PSMD4, UBA3, KEAP1, HLA-C, UBE2L3, FBXL5, PSMD6, SOCS1, CDC27, UBE2M, UBE2U, KLHL11, UBE2B, KLHL3, MRC2, TLR2, UBE2E2, RCHY1, FZR1, UBE2L6, LMO7, FBXO7, PSMA7, PSME4, FBXO10, UBE2H, RNF19A, SEC23A, FBXW10, CTSS, NCF1, RNF126, ZNRF2, PSMB1, NPEPPS, UBR2, KLHL22, ASB6, SIAH2, PSMD7, FBXO9, SMURF1, CD207, FBXW8, UBE2O, FBXL20, ASB2, KCTD6, TAP1, PSMF1, UBE2R2, UBE2Z, PSMD11, MYLIP, HERC4, VAMP8, RNF111, IKBKB, FBXO30, HERC2, RNF34, FBXL3, PSMD3, PSMA6, PSMD9, HERC1, ASB18, VAMP3, KLHL9, CDC34, DZIP3, FBXL12, SEC24C, ANAPC5, CD36, SKP1, CD14, CDC26, TRIM50, PDIA3, RNF182, WWP1, PSMB9, FBXO15, FBXL22, TIRAP, NEDD4L, HERC3, FBXL18* |
| REACTOME_R_HSA_264876 | Insulin processing | 7.75E-03 | 7739 | 23 | ***EXOC3****, EXOC4, VAMP2, MYO5A* |
| REACTOME_R_HSA_1445148 | Translocation of GLUT4 to the plasma membrane | 7.82E-03 | 7817 | 52 | ***EXOC3****, EXOC4, PRKAB1, KIF3B, VAMP2, MYO5A, CALM1, RAB4A, ASPSCR1, CALM3, RAB13, EXOC8* |
| REACTOME_R_HSA_5678895 | Defective CFTR causes cystic fibrosis | 8.22E-03 | 8216 | 39 | *PSMD4, RNF5, CFTR, PSMD6, ERLEC1, PSMA7, VCP, PSME4, DERL1, PSMB1, PSMD7, PSMF1, PSMD11, PSMD3, PSMA6, PSMD9* |
| BIOCYC_HUMAN_PWY_5695 | urate biosynthesis/inosine 5'-phosphate degradation | 8.81E-03 | 7751 | 5 | *IMPDH1, NT5C2* |
| Pathway Interaction Database_il12_stat4pathway | IL12 signaling mediated by STAT4 | 9.38E-03 | 9318 | 16 | ***HLA-DRB1****, HLA-DRA, TGFB1, PIAS2, IL13, MAPK9, CD4* |
| KEGG_hsa05206 | MicroRNAs in cancer | 9.81E-03 | 9814 | 110 | *MET, NOTCH4,* ***PRKCB****, DNMT3B, CD44, ST14, DDIT4, TNXB, BCL2, SOCS1, FZD3, NOTCH1, RDX, SOX4, ERBB2, KRAS, STMN1, IRS1, BMPR2, CCNE2, ABCB1, TP63, PLAU, VEGFA, EP300, GRB2, CCNG1, ZEB1, SOS2, MARCKS, SPRY2, IKBKB, PDCD4, CDC25C, TGFB2, PIK3R2, TPM1, ROCK1, PIK3CA, WNT3, CRKL, SOS1, MAP2K2, ABL1* |

**S3 Table**. **Top 30 KNoRMA associated candidate modifiers from GTEx Lung imputed gene expression**

Association of imputed gene expression from GTEx lung to KNoRMA were tested by robust linear regression using LIMMA.

**SNPs in model (*n*)** = number of SNPs used in the predictive model for gene expression imputation

**Var Expl by SNPs** = variance (of gene expression) explained by SNPs in the model, or R^2^ between predicted and observed gene expression

**Adjusted P value** = FDR adjusted p-value

| **Gene*** | **Description** | **Gene type** | **Chr** | **SNPs in model (n)** | **Var Expl by SNPs** | **t** | **P value** | **Adjusted P value** |
| --- | --- | --- | --- | --- | --- | --- | --- | --- |
| ***ZDHHC11B*** | zinc finger, DHHC-type containing 11B | protein_coding | 5 | 21 | 0.16 | 6.04 | 1.62E-09 | 1.28E-05 |
| ***HLA-DRB6*** | major histocompatibility complex, class II, DR beta 6 (pseudogene) | pseudogene | 6 | 54 | 0.67 | -5.44 | 5.42E-08 | 2.15E-04 |
| ***HLA-DQA2*** | major histocompatibility complex, class II, DQ alpha 2 | protein_coding | 6 | 49 | 0.65 | -5.00 | 5.80E-07 | 1.53E-03 |
| ***EXOC3*** | exocyst complex component 3 | protein_coding | 5 | 13 | 0.24 | 4.94 | 8.17E-07 | 1.62E-03 |
| ***HLA-DQA1*** | major histocompatibility complex, class II, DQ alpha 1 | protein_coding | 6 | 32 | 0.52 | 4.89 | 1.05E-06 | 1.67E-03 |
| ***HLA-DRB1*** | major histocompatibility complex, class II, DR beta 1 | protein_coding | 6 | 32 | 0.36 | 4.57 | 4.99E-06 | 6.59E-03 |
| *MXRA7* | matrix-remodelling associated 7 | protein_coding | 17 | 25 | 0.60 | -4.15 | 3.43E-05 | 3.83E-02 |
| *PDLIM1P4* | PDZ and LIM domain 1 pseudogene 4 | pseudogene | 3 | 29 | 0.03 | -4.12 | 3.87E-05 | 3.83E-02 |
| ***SDHAP1*** | succinate dehydrogenase complex, subunit A, flavoprotein pseudogene 1 | pseudogene | 3 | 51 | 0.22 | -3.96 | 7.67E-05 | 6.44E-02 |
| *TDRKH* | tudor and KH domain containing | protein_coding | 1 | 17 | 0.10 | -3.94 | 8.11E-05 | 6.44E-02 |
| *TMPRSS6* | transmembrane protease, serine 6 | protein_coding | 22 | 21 | 0.12 | -3.75 | 1.75E-04 | 1.26E-01 |
| *ATP6V0E2* | ATPase, H+ transporting V0 subunit e2 | protein_coding | 7 | 7 | 0.25 | -3.73 | 1.94E-04 | 1.28E-01 |
| ***HLA-DQB1*** | major histocompatibility complex, class II, DQ beta 1 | protein_coding | 6 | 40 | 0.63 | 3.69 | 2.22E-04 | 1.36E-01 |
| *RP3-462E2.5* |  | lincRNA | 12 | 20 | 0.08 | 3.63 | 2.82E-04 | 1.45E-01 |
| *IBTK* | inhibitor of Bruton agammaglobulinemia tyrosine kinase | protein_coding | 6 | 46 | 0.02 | 3.63 | 2.85E-04 | 1.45E-01 |
| *HLA-DQB2* | major histocompatibility complex, class II, DQ beta 2 | protein_coding | 6 | 39 | 0.52 | -3.61 | 3.11E-04 | 1.45E-01 |
| *SMTNL1* | smoothelin-like 1 | protein_coding | 11 | 37 | 0.40 | 3.61 | 3.11E-04 | 1.45E-01 |
| *AC026740.1* | Uncharacterized protein | protein_coding | 5 | 35 | 0.15 | 3.53 | 4.13E-04 | 1.75E-01 |
| *MYPN* | myopalladin | protein_coding | 10 | 14 | 0.02 | -3.51 | 4.48E-04 | 1.75E-01 |
| *LDLRAP1* | low density lipoprotein receptor adaptor protein 1 | protein_coding | 1 | 13 | 0.34 | 3.50 | 4.62E-04 | 1.75E-01 |
| *SLC6A12* | solute carrier family 6 (neurotransmitter transporter), member 12 | protein_coding | 12 | 3 | 0.03 | 3.50 | 4.63E-04 | 1.75E-01 |
| *DESI1* | desumoylating isopeptidase 1 | protein_coding | 22 | 11 | 0.08 | -3.43 | 6.03E-04 | 2.11E-01 |
| *APOA1BP* | apolipoprotein A-I binding protein | protein_coding | 1 | 12 | 0.04 | -3.43 | 6.13E-04 | 2.11E-01 |
| *APOC2* | apolipoprotein C-II | protein_coding | 19 | 11 | 0.13 | -3.37 | 7.59E-04 | 2.51E-01 |
| *HEATR2* | HEAT repeat containing 2 | protein_coding | 7 | 37 | 0.28 | 3.32 | 9.03E-04 | 2.86E-01 |
| *ARFGAP3* | ADP-ribosylation factor GTPase activating protein 3 | protein_coding | 22 | 25 | 0.13 | -3.29 | 9.98E-04 | 3.05E-01 |
| *ORMDL3* | ORM1-like 3 (S. cerevisiae) | protein_coding | 17 | 8 | 0.14 | -3.28 | 1.05E-03 | 3.09E-01 |
| *EDEM3* | ER degradation enhancer, mannosidase alpha-like 3 | protein_coding | 1 | 13 | 0.30 | 3.25 | 1.18E-03 | 3.34E-01 |
| *C22orf39* | chromosome 22 open reading frame 39 | protein_coding | 22 | 22 | 0.06 | -3.22 | 1.30E-03 | 3.46E-01 |
| *NUDT16P* | nudix (nucleoside diphosphate linked moiety X)-type motif 16 pseudogene 1 | pseudogene | 3 | 6 | 0.02 | -3.21 | 1.32E-03 | 3.46E-01 |

*** *bold***: consensus genes near GWAS loci; *underlined*: consensus genes outside GWAS loci

**S4 Table**. **Top 30 KNoRMA associated candidate modifiers from CF nasal epithelium imputed gene expression**

Association of imputed gene expression from CF nasal epithelium to KNoRMA were tested by robust linear regression using LIMMA.

**SNPs in model (*n*)** = number of SNPs used in the predictive model for gene expression imputation

**Var Expl by SNPs** = variance (of gene expression) explained by SNPs in the model, or R^2^ between predicted and observed gene expression

**Adjusted P value** = FDR adjusted p-value

| **Gene*** | **Description** | **Gene type** | **Chr** | **SNPs in model (n)** | **Var Expl by SNPs** | ***t*** | **P value** | **Adjusted P value** |
| --- | --- | --- | --- | --- | --- | --- | --- | --- |
| *ARFGAP3* | ADP ribosylation factor GTPase activating protein 3 | protein_coding | 22 | 28 | 0.23 | -5.55 | 2.96E-08 | 8.53E-05 |
| *SDHAP2* | succinate dehydrogenase complex flavoprotein subunit A pseudogene 2 | transcribed_unprocessed_pseudogene | 3 | 10 | 0.05 | 4.45 | 8.56E-06 | 1.23E-02 |
| ***HLA-DQA2*** | major histocompatibility complex, class II, DQ alpha 2 | protein_coding | 6 | 44 | 0.39 | -3.94 | 8.36E-05 | 5.73E-02 |
| ***HLA-DRB9*** | major histocompatibility complex, class II, DR beta 9 (pseudogene) | unprocessed_pseudogene | 6 | 29 | 0.22 | -3.91 | 9.42E-05 | 5.73E-02 |
| *FANCG* | Fanconi anemia complementation group G | protein_coding | 9 | 4 | 0.10 | -3.86 | 1.13E-04 | 5.73E-02 |
| *ENTPD2* | ectonucleoside triphosphate diphosphohydrolase 2 | protein_coding | 9 | 35 | 0.04 | 3.85 | 1.19E-04 | 5.73E-02 |
| *GLRX* | glutaredoxin | protein_coding | 5 | 23 | 0.07 | -3.54 | 4.00E-04 | 1.64E-01 |
| ***HLA-DRB1*** | major histocompatibility complex, class II, DR beta 1 | protein_coding | 6 | 43 | 0.11 | 3.29 | 1.01E-03 | 3.65E-01 |
| *KRT31* | keratin 31 | protein_coding | 17 | 9 | 0.10 | -3.22 | 1.28E-03 | 4.01E-01 |
| *BPIFA1* | BPI fold containing family A member 1 | protein_coding | 20 | 14 | 0.49 | 3.20 | 1.39E-03 | 4.01E-01 |
| *GOLGA8Q* | golgin A8 family member Q | protein_coding | 15 | 15 | 0.04 | -3.15 | 1.66E-03 | 4.34E-01 |
| ***HLA-DQB1*** | major histocompatibility complex, class II, DQ beta 1 | protein_coding | 6 | 48 | 0.61 | 3.08 | 2.07E-03 | 4.65E-01 |
| ***SDHAP1*** | succinate dehydrogenase complex flavoprotein subunit A pseudogene 1 | transcribed_unprocessed_pseudogene | 3 | 43 | 0.22 | -3.07 | 2.15E-03 | 4.65E-01 |
| *NDUFA3* | NADH:ubiquinone oxidoreductase subunit A3 | protein_coding | 19 | 37 | 0.15 | -3.05 | 2.26E-03 | 4.65E-01 |
| *C8orf22* | chromosome 8 open reading frame 22 | protein_coding | 8 | 58 | 0.24 | 3.03 | 2.46E-03 | 4.72E-01 |
| *SCIN* | scinderin | protein_coding | 7 | 3 | 0.07 | 2.99 | 2.79E-03 | 5.02E-01 |
| *SEPT3* | septin 3 | protein_coding | 22 | 10 | 0.04 | -2.97 | 3.01E-03 | 5.11E-01 |
| *SPATA31B1P* | SPATA31 subfamily B member 1, pseudogene | unprocessed_pseudogene | 9 | 5 | 0.06 | -2.92 | 3.52E-03 | 5.50E-01 |
| *DOCK9* | dedicator of cytokinesis 9 | protein_coding | 13 | 18 | 0.04 | 2.91 | 3.63E-03 | 5.50E-01 |
| *NIPA2P1* | non imprinted in Prader-Willi/Angelman syndrome 2 pseudogene 1 | processed_pseudogene | 7 | 22 | 0.07 | 2.86 | 4.21E-03 | 6.06E-01 |
| *FAM173A* | family with sequence similarity 173 member A | protein_coding | 16 | 16 | 0.05 | 2.84 | 4.55E-03 | 6.14E-01 |
| *BMI1* | BMI1 proto-oncogene, polycomb ring finger | protein_coding | 10 | 7 | 0.05 | 2.81 | 4.90E-03 | 6.14E-01 |
| ***HLA-DQA1*** | major histocompatibility complex, class II, DQ alpha 1 | protein_coding | 6 | 44 | 0.39 | 2.81 | 4.90E-03 | 6.14E-01 |
| *ZNF526* | zinc finger protein 526 | protein_coding | 19 | 16 | 0.05 | 2.77 | 5.60E-03 | 6.51E-01 |
| *STRA6* | stimulated by retinoic acid 6 | protein_coding | 15 | 8 | 0.13 | 2.77 | 5.70E-03 | 6.51E-01 |
| *SNRPCP16* | small nuclear ribonucleoprotein polypeptide C pseudogene 16 | processed_pseudogene | 4 | 23 | 0.07 | -2.75 | 6.01E-03 | 6.51E-01 |
| ***MUC20*** | mucin 20, cell surface associated | protein_coding | 3 | 33 | 0.28 | -2.74 | 6.11E-03 | 6.51E-01 |
| *HRCT1* | histidine rich carboxyl terminus 1 | protein_coding | 9 | 59 | 0.08 | 2.70 | 6.94E-03 | 6.73E-01 |
| *CFHR4* | complement factor H related 4 | protein_coding | 1 | 32 | 0.18 | 2.69 | 7.10E-03 | 6.73E-01 |
| *HSF2* | heat shock transcription factor 2 | protein_coding | 6 | 44 | 0.08 | -2.69 | 7.18E-03 | 6.73E-01 |

*** *bold***: consensus genes near GWAS loci; *underlined*: consensus genes outside GWAS loci

**S1 File. Candidate CF lung disease modifier genes (379)**. Gene annotation from ENSEMBL v90, GO associations, and multi-tissue meta-analysis p-values and mean effect sizes from CF lung function association testing for 379 candidate modifier genes are contained in Microsoft Excel file.

**S2 File. CF lung function (KNoRMA) association testing for imputed gene expression based on predictive models derived from CF nasal epithelial biopsy gene expression data set**. Gene annotation from ENSEMBL v90, selected predictive model performance metrics, and imputed gene expression association (to KNoRMA) testing results are stored in Microsoft Excel file.

**S3 File. CF lung function association testing for imputed gene expression from predictive models derived from alternative mapping strategy**. Similar results as supplementary S2 File, except the predictive models were derived from gene expression estimation from alternative mapping strategy of RNA-seq reads, against multiple alternative assemblies, and accounting for variants from dbSNP v150.

**References**

1. Panjwani N, Xiao B, Xu L, Gong J, Keenan K, Lin F, et al. Improving imputation in disease-relevant regions: lessons from cystic fibrosis. NPJ Genom Med. 2018;3:8. Epub 2018/03/28. doi: 10.1038/s41525-018-0047-6. PubMed PMID: 29581887; PubMed Central PMCID: PMCPMC5861096.

2. Gong J, Wang F, Xiao B, Panjwani N, Lin F, Keenan K, et al. Genetic association and transcriptome integration identify contributing genes and tissues at cystic fibrosis modifier loci. PLoS Genet. 2019;15(2):e1008007. Epub 2019/02/27. doi: 10.1371/journal.pgen.1008007. PubMed PMID: 30807572.

3. Polineni D, Dang H, Gallins PJ, Jones LC, Pace RG, Stonebraker JR, et al. Airway Mucosal Host Defense Is Key to Genomic Regulation of Cystic Fibrosis Lung Disease Severity. Am J Respir Crit Care Med. 2018;197(1):79-93. Epub 2017/08/31. doi: 10.1164/rccm.201701-0134OC. PubMed PMID: 28853905; PubMed Central PMCID: PMCPMC5765386.

4. Kim D, Langmead B, Salzberg SL. HISAT: a fast spliced aligner with low memory requirements. Nat Methods. 2015;12(4):357-60. Epub 2015/03/10. doi: 10.1038/nmeth.3317. PubMed PMID: 25751142; PubMed Central PMCID: PMCPMC4655817.

5. Pertea M, Pertea GM, Antonescu CM, Chang TC, Mendell JT, Salzberg SL. StringTie enables improved reconstruction of a transcriptome from RNA-seq reads. Nat Biotechnol. 2015;33(3):290-5. Epub 2015/02/19. doi: 10.1038/nbt.3122. PubMed PMID: 25690850; PubMed Central PMCID: PMCPMC4643835.

6. Lee W, Plant K, Humburg P, Knight JC. AltHapAlignR: improved accuracy of RNA-seq analyses through the use of alternative haplotypes. Bioinformatics. 2018. Epub 2018/03/08. doi: 10.1093/bioinformatics/bty125. PubMed PMID: 29514179.

7. O'Neal WK, Gallins P, Pace RG, Dang H, Wolf WE, Jones LC, et al. Gene expression in transformed lymphocytes reveals variation in endomembrane and HLA pathways modifying cystic fibrosis pulmonary phenotypes. Am J Hum Genet. 2015;96(2):318-28. Epub 2015/02/03. doi: 10.1016/j.ajhg.2014.12.022. PubMed PMID: 25640674; PubMed Central PMCID: PMCPMC4320265.

8. Gamazon ER, Wheeler HE, Shah KP, Mozaffari SV, Aquino-Michaels K, Carroll RJ, et al. A gene-based association method for mapping traits using reference transcriptome data. Nat Genet. 2015;47(9):1091-8. Epub 2015/08/11. doi: 10.1038/ng.3367. PubMed PMID: 26258848; PubMed Central PMCID: PMCPMC4552594.

9. Friedman J, Hastie T, Tibshirani R. Regularization Paths for Generalized Linear Models via Coordinate Descent. J Stat Softw. 2010;33(1):1-22. Epub 2010/09/03. PubMed PMID: 20808728; PubMed Central PMCID: PMCPMC2929880.

10. Gusev A, Ko A, Shi H, Bhatia G, Chung W, Penninx BW, et al. Integrative approaches for large-scale transcriptome-wide association studies. Nat Genet. 2016;48(3):245-52. Epub 2016/02/09. doi: 10.1038/ng.3506. PubMed PMID: 26854917; PubMed Central PMCID: PMCPMC4767558.

11. Consortium GT. The Genotype-Tissue Expression (GTEx) project. Nat Genet. 2013;45(6):580-5. Epub 2013/05/30. doi: 10.1038/ng.2653. PubMed PMID: 23715323; PubMed Central PMCID: PMCPMC4010069.

12. Battle A, Mostafavi S, Zhu X, Potash JB, Weissman MM, McCormick C, et al. Characterizing the genetic basis of transcriptome diversity through RNA-sequencing of 922 individuals. Genome Res. 2014;24(1):14-24. Epub 2013/10/05. doi: 10.1101/gr.155192.113. PubMed PMID: 24092820; PubMed Central PMCID: PMCPMC3875855.

13. Corvol H, Blackman SM, Boelle PY, Gallins PJ, Pace RG, Stonebraker JR, et al. Genome-wide association meta-analysis identifies five modifier loci of lung disease severity in cystic fibrosis. Nat Commun. 2015;6:8382. Epub 2015/09/30. doi: 10.1038/ncomms9382. PubMed PMID: 26417704; PubMed Central PMCID: PMCPMC4589222.

14. Wright FA, Strug LJ, Doshi VK, Commander CW, Blackman SM, Sun L, et al. Genome-wide association and linkage identify modifier loci of lung disease severity in cystic fibrosis at 11p13 and 20q13.2. Nat Genet. 2011;43(6):539-46. Epub 2011/05/24. doi: 10.1038/ng.838. PubMed PMID: 21602797; PubMed Central PMCID: PMCPMC3296486.

15. Pruim RJ, Welch RP, Sanna S, Teslovich TM, Chines PS, Gliedt TP, et al. LocusZoom: regional visualization of genome-wide association scan results. Bioinformatics. 2010;26(18):2336-7. Epub 2010/07/17. doi: 10.1093/bioinformatics/btq419. PubMed PMID: 20634204; PubMed Central PMCID: PMCPMC2935401.

16. Ritchie ME, Phipson B, Wu D, Hu Y, Law CW, Shi W, et al. limma powers differential expression analyses for RNA-sequencing and microarray studies. Nucleic Acids Res. 2015;43(7):e47. Epub 2015/01/22. doi: 10.1093/nar/gkv007. PubMed PMID: 25605792; PubMed Central PMCID: PMCPMC4402510.

17. Marazzi A, Joss J, Randriamiharisoa A. Algorithms, routines, and S functions for robust statistics : the FORTRAN library ROBETH with an interface to S-PLUS. Pacific Grove, Calif.: Wadsworth & Brooks/Cole Advanced Books & Software; 1993. xii, 436 p. p.

18. Venables WN, Ripley BD, Venables WN. Modern applied statistics with S. 4th ed. New York: Springer; 2002. xi, 495 p. p.

19. Gu Z, Eils R, Schlesner M. Complex heatmaps reveal patterns and correlations in multidimensional genomic data. Bioinformatics. 2016;32(18):2847-9. Epub 2016/05/22. doi: 10.1093/bioinformatics/btw313. PubMed PMID: 27207943.

20. Turner SD. qqman: an R package for visualizing GWAS results using Q-Q and manhattan plots. bioRxiv. 2014. doi: 10.1101/005165.

21. Hadley W. Ggplot2. New York, NY: Springer Science+Business Media, LLC; 2016. pages cm p.

22. Bulik-Sullivan BK, Loh PR, Finucane HK, Ripke S, Yang J, Schizophrenia Working Group of the Psychiatric Genomics C, et al. LD Score regression distinguishes confounding from polygenicity in genome-wide association studies. Nat Genet. 2015;47(3):291-5. Epub 2015/02/03. doi: 10.1038/ng.3211. PubMed PMID: 25642630; PubMed Central PMCID: PMCPMC4495769.

23. Wilson DJ. The harmonic mean p-value for combining dependent tests. bioRxiv. 2018. doi: 10.1101/171751.

24. Poole W, Gibbs DL, Shmulevich I, Bernard B, Knijnenburg TA. Combining dependent P-values with an empirical adaptation of Brown's method. Bioinformatics. 2016;32(17):i430-i6. Epub 2016/09/03. doi: 10.1093/bioinformatics/btw438. PubMed PMID: 27587659; PubMed Central PMCID: PMCPMC5013915.

25. Sergushichev A. An algorithm for fast preranked gene set enrichment analysis using cumulative statistic calculation. bioRxiv. 2016:060012. doi: 10.1101/060012.

26. Ashburner M, Ball CA, Blake JA, Botstein D, Butler H, Cherry JM, et al. Gene ontology: tool for the unification of biology. The Gene Ontology Consortium. Nat Genet. 2000;25(1):25-9. Epub 2000/05/10. doi: 10.1038/75556. PubMed PMID: 10802651; PubMed Central PMCID: PMCPMC3037419.

27. Geer LY, Marchler-Bauer A, Geer RC, Han L, He J, He S, et al. The NCBI BioSystems database. Nucleic Acids Res. 2010;38(Database issue):D492-6. Epub 2009/10/27. doi: 10.1093/nar/gkp858. PubMed PMID: 19854944; PubMed Central PMCID: PMCPMC2808896.

28. Subramanian A, Tamayo P, Mootha VK, Mukherjee S, Ebert BL, Gillette MA, et al. Gene set enrichment analysis: a knowledge-based approach for interpreting genome-wide expression profiles. Proc Natl Acad Sci U S A. 2005;102(43):15545-50. Epub 2005/10/04. doi: 10.1073/pnas.0506580102. PubMed PMID: 16199517; PubMed Central PMCID: PMCPMC1239896.
